# Polycomb Group Gene *OsFIE2* Regulates Rice (*Oryza sativa*) Seed Development and Grain Filling via a Mechanism Distinct from *Arabidopsis*

**DOI:** 10.1371/journal.pgen.1003322

**Published:** 2013-03-07

**Authors:** Babi Ramesh Reddy Nallamilli, Jian Zhang, Hana Mujahid, Brandon M. Malone, Susan M. Bridges, Zhaohua Peng

**Affiliations:** 1 Department of Biochemistry and Molecular Biology, Mississippi State University, Mississippi State, Mississippi, United States of America; 2 Department of Computer Science and Engineering, Mississippi State University, Mississippi State, Mississippi, United States of America; 3 Institute for Genomics, Biocomputing and Biotechnology, Mississippi State University, Mississippi State, Mississippi, United States of America; Peking University, China

## Abstract

Cereal endosperm represents 60% of the calories consumed by human beings worldwide. In addition, cereals also serve as the primary feedstock for livestock. However, the regulatory mechanism of cereal endosperm and seed development is largely unknown. Polycomb complex has been shown to play a key role in the regulation of endosperm development in *Arabidopsis*, but its role in cereal endosperm development remains obscure. Additionally, the enzyme activities of the polycomb complexes have not been demonstrated in plants. Here we purified the rice *OsFIE2*-polycomb complex using tandem affinity purification and demonstrated its specific H3 methyltransferase activity. We found that the *OsFIE2* gene product was responsible for H3K27me3 production specifically *in vivo*. Genetic studies showed that a reduction of *OsFIE2* expression led to smaller seeds, partially filled seeds, and partial loss of seed dormancy. Gene expression and proteomics analyses found that the starch synthesis rate limiting step enzyme and multiple storage proteins are down-regulated in *OsFIE2* reduction lines. Genome wide ChIP–Seq data analysis shows that H3K27me3 is associated with many genes in the young seeds. The H3K27me3 modification and gene expression in a key helix-loop-helix transcription factor is shown to be regulated by *OsFIE2*. Our results suggest that *OsFIE2*-polycomb complex positively regulates rice endosperm development and grain filling via a mechanism highly different from that in *Arabidopsis*.

## Author Summary

Rice is the staple food for over half of the world's population and an important feedstock for livestock. The rice grain is mainly endosperm tissue. The regulatory mechanism of rice endosperm development is still largely unknown thus far. Understanding the underlying mechanism will lead to crop yield and quality improvement in the long term, besides gaining new knowledge. Polycomb complex is a protein complex with a potential role in endosperm development according to prior publications. In this manuscript, we purified the rice OsFIE2-polycomb protein complex and demonstrated the enzyme activity of the complex. Genetic studies showed that a reduction of polycomb group gene *OsFIE2* expression led to smaller seeds, partially filled seeds, and seed germination before seed maturation. Gene expression and proteomics analyses found that the starch synthesis rate limiting step enzyme and multiple storage proteins are down-regulated while a key transcription factor is up-regulated in *OsFIE2* reduction lines. In addition, we identified many loci in the rice genome whose histone proteins are modified by the polycomb complex enzyme via a method called ChIP–Seq. Our results demonstrate that OsFIE2-polycomb complex positively regulates rice grain development via a mechanism distinct from that in *Arabidopsis* and provide new insight into the regulation of rice grain development.

## Introduction

Rice (*Oryza sativa*) serves as the staple food for over half of the world's population. The main body of rice grain is endosperm, which is consumed as food. Endosperm stores energy primarily in the form of starch, storage proteins, and lipids. As in other angiosperms, rice seed development requires initiation signals from double fertilization. During double fertilization, one sperm cell fuses with the egg cell to develop into the diploid embryo while the other sperm cell fuses with the central cell of the female gametophyte to develop into the triploid endosperm. Before fertilization, premature divisions of the egg and the central cells are suppressed. Substantial progress has been made in understanding the role of the polycomb group (PcG) genes in the repression of central cell division before fertilization in *Arabidopsis*.

Polycomb group (PcG) genes, first discovered in *Drosophila melanogaster*, play an important role in maintaining the repressed state of homeotic (HOX) genes by posttranslational modifications of histones [1]. PcG genes also control a large number of genes regulating many cellular functions and developmental pathways, such as cell proliferation, stem cell maintenance, imprinting and cancer [2], [3]. PcG proteins form three principle types of multiprotein complexes, Polycomb Repressive Complex 1 (PRC1), Polycomb Repressive Complex 2 (PRC2) and Pho RC. PcG complexes have been identified in different organisms, including *Drosophila melanogaster* and *Arabidopsis thaliana* [3]–[9]. In *Drosophila melanogaster*, PRC2 complex consists of E (Z), ESC, Su (Z) 12 and p55 [10].

The identification and characterization of PRC2 complex genes in plants indicated remarkable structural and functional conservation of the PRC2 complexes between plants and animals. Genetic and molecular studies suggested the presence of three PRC2-like complexes in *Arabidopsis thaliana*: the Fertilization Independent Seed (FIS), Embryonic Flower (EMF) and Vernalization (VRN) complexes [3], [8], [9], [11]–[17]. The EMF complex, which probably contains CLF/SWN, EMF2, FIE and MSI1, promotes vegetative development by repressing the transcription of flowering activators such as *Flowering Locus T* (*FT*) and *Agamous-like 19* (*AGL19*) [18]–[20]. The VRN complex is involved in cold-induced epigenetic silencing of *Arabidopsis Flowering locus C* (*FLC*). The VRN complex is purified biochemically by tandem affinity purification method [3]. It includes *VRN2*, *SWINGER* (E (Z) homolog), *FIE* (ESC homolog), *MSI1* (p55 homolog), *VRN5*, *VIN3*, and *VEL1* [3], [4], [13], [14], [21], [22]. The VRN complex associates with PHD-finger proteins for better H3K27me3 deposition. Mathematical modeling showed that a polycomb-based switch underlying quantitative epigenetic memory [23].

Mutations of *FIS* complex genes (*MEA*, *FIS2*, *FIE* and *MSI1*) result in the formation of multinucleate central cell that develops to the point of cellularization in the absence of double fertilization. The partially developed seed like structures eventually atrophy. Multinucleate central cell that develops to an endosperm was capable of nourishing an embryo [24]. Mutations in *Arabidopsis FIS* genes also show an interesting phenotype after fertilization, including endosperm over-proliferation, embryo arrest and abortion. In *FIS* mutants, endosperm nuclei continue to divide even after the wild type endosperm stopped to replicate and develops large balloon like seeds with delayed cellularization in the endosperm. Gradually, the endosperm collapses and the seed aborts. It is not clear whether the endosperm and embryo phenotypes are both directly due to the *FIS* gene mutation or whether one is the primary defect and the other is a downstream event [25].

Mutations in *Arabidopsis FIS* genes also show parent-of-origin effect on seed development [5]–[8], [26]. Every seed that inherits a maternal mutant *FIS* allele aborts regardless of the presence of a wild type paternal allele [5], [27]–[30]. *MEADEA* (*FIS1*) and *FIS2* are imprinted genes and expressed maternally throughout endosperm development in *Arabidopsis* [5], [31]–[33]. *FIE* (*FIS3*) maternal allele is expressed in the early endosperm [12], [16]. A recent study using the *FIE* mutant showed that the PRC2 complex is essential for the transition from embryonic phase to the seedling stage [34]. The *FIE* mutant show delayed germination. 40% of the homozygous *FIE* mutants failed to germinate after 20 days while the wildtype germinated within 2 days. In addition, polycomb group proteins are required to couple seed coat initiation to fertilization [35].

Cereal crops, such as Maize, barley and Rice, have multiple homologs of PRC2 core complex genes. Two homologs of *ESC* are identified in maize, *ZmFie1* and *ZmFie2*. The *ZmFie1* is expressed only in endosperm and imprinted during endosperm development, whereas *ZmFie2* is expressed in the egg cell and more intensively in the central cell [36]–[38]. Maize has three *E (Z)* homologs: *MEZ1*, *MEZ2*, and *MEZ3* [39], [40]. Four polycomb gene homologs are identified in barley, *HvFIE*, *HvE(Z)*, *HvSu(Z)12a* and *HvSu(Z)12b* [41]. Genome wide analysis studies indicated the presence of multiple homologs of *ESC*, *E (Z)*, and *Su (Z) 12* in Rice genome [42]. Rice has two *ESC* homologs (*OsFIE1* (Os08g04290) and *OsFIE2* (Os08g04270)), two *E (Z)* homologs (*OsSET1* (Os03g19480) and *OsCLF* (Os06g16390)) and two *Su (Z) 12* homologs (*OsEMF2a* (Os04g08034) and *OsEMF2b* (Os09g13630)) [42]–[45].

Interestingly, not all the FIS complex functions identified in *Arabidopsis* are conserved in other plants [42], [46]. Both imprinted genes *MEA* and *FIS2* are involved in the central cell repression and endosperm development in *Arabidopsis*. But rice, maize and barley genomes do not have *MEA* and *FIS2* gene orthologs [42], [46], [47]. In addition, all the reported rice polycomb genes are widely expressed in different tissues including endosperm except that *OsFIE1* is imprinted and the maternal allele is expressed specifically in endosperm [42], [45]. Mutations in *Arabidopsis* FIS genes show autonomous endosperm development whereas T-DNA insertion line in *OsFIE1* gene did not result in autonomous central cell proliferation in rice [42], suggesting that even though *OsFIE1* is imprinted in rice endosperm, it is not involved in the repression of central cell proliferation. Knockdown of *ZmFIE1* and *ZmFIE2* genes in maize also produced no autonomous central cell proliferation [46]. In sexual *Hieracium pilosella*, RNAi lines of *HFIE* failed to show the autonomous central cell proliferation [48]. But down-regulation of *HFIE* in sexual *Hieracium* resulted in seed abortion after fertilization. Similarly, specific *HFIE* down regulation in the apomictic *Hieracium* resulted in embryo abortion and defective endosperm, indicating that *HFIE* gene is important for the development of viable seed in both sexual and apomictic *Hieracium* [48]. The presence of homologs of *Arabidopsis* polycomb genes in rice, maize and barley genomes indicates the conservation of polycomb genes between monocot and dicot plants. Whereas, the lack of some important seed specific *FIS* genes (*MEA* and *FIS2*) in rice, maize and barley genomes suggests that there might be different functional regulatory mechanisms involved in the embryo and endosperm development in monocots.

Despite the extensive genetic studies on polycomb complex genes during the seed development in plants, biochemical characterization of the polycomb complex is still very limited except that the VRN complex has been purified via tandem affinity purification in *Arabidopsis*. The enzyme activities of the PcG complex have not been demonstrated *in vitro* for plants. To elucidate the biological function of the polycomb complex and its molecular mechanism in the regulation of gene expression, we purified the polycomb complex, identified the components of the complex via mass spectrometry analysis, and detected the enzyme activity of the complex by *in vitro* assay. In addition, we generated RNAi and over expression lines of *OsFIE2*. The phenotype of the mutant lines clearly demonstrated an essential role of the complex in the regulation of endosperm development, grain filling and seed dormancy. The regulatory mechanisms are different from those in *Arabidopsis*. Transcription and proteomic analysis of selected nutrient metabolic pathway genes showed that many of them are subject to *OsFIE2* regulation, directly or indirectly. Finally, the H3K27me3 binding sites in young endosperm were identified using ChIP-Seq approach. Our results suggest that the polycomb group genes control seed development and grain filling by regulating a large number of genes.

## Results

### Tandem affinity purification of *OsFIE2*-interacting proteins

**Figure 1:**
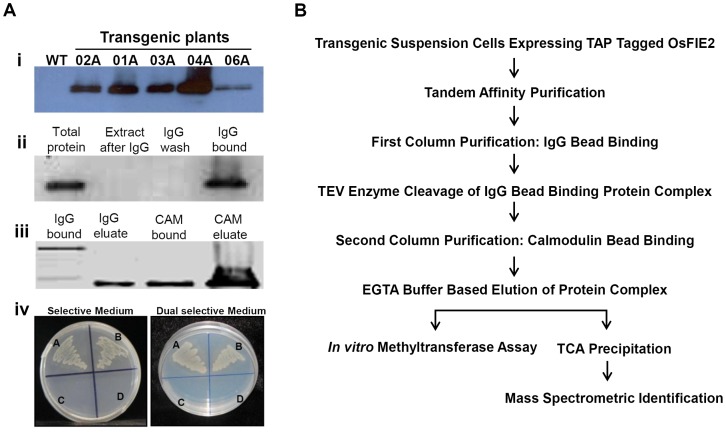
Tandem affinity purification of OsFIE2 complex. (A) Western blot analyses and *E.coli* two hybrid assay. (i) TAP tagged *OsFIE2* expression in different transgenic lines. The transgenic lines are indicated on the top. Antibodies specific for the protein A domain of the TAP tag were used for Western blots shown in panels i and ii. (ii) Western blot analysis of the samples collected in the first column purification phase. (iii) Western blot analysis of the samples collected in the second column purification phase. CBP domain was detected by using biotinylated CAM [51]. (iv) *E. coli* two hybrid assay. A: pBT-LGF2+pTRG-Gal II - Positive control. B: Recombinant pBT-*OsSET1*+ Recombinant pTRG*-OsFIE2*. C: Recombinant pBT-*OsSET1*+ Empty pTRG - Negative control. D: Empty pBT+Recombinant pTRG-*OsFIE2* - Negative control. Co-transformations were performed with above mentioned pair wise plasmid combinations. Positive interactions were identified by screening co-transformed cells on selective medium containing 3-Amino-1, 2, 4-triazole (3-AT). Positive interactions were further confirmed by screening on dual selective medium containing 3-AT and streptomycin. (B) Schematic representation of different steps used in tandem affinity purification of OsFIE2 complex.

The FIE proteins belong to a family of WD-repeat proteins that promote protein-protein interaction in various multiprotein complexes. They are necessary for the viable seed formation in both *Arabidopsis* and *Hieracium*. To identify the proteins associated with rice OsFIE2, the tandem affinity purification (TAP) approach was used [3], [49]–[57]. The TAP tag used in this study contains protein A, the TEV recognition site and the calmodulin-binding protein (CBP) domain [49]–[51]. The final N-Terminal TAP-Tagged transgenic rice plants and cell suspension cultures expressing the TAP-Tagged OsFIE2 were generated. The expression of TAP-tagged OsFIE2 protein was verified by Western blots in multiple transgenic lines (Figure 1A-i). A cell culture system established from the most highly expressed transgenic line 04A was selected for future studies.

Different steps invloved in the TAP purification method are depicted in Figure 1B. In the first step of the tandem purification, protein A domain of OsFIE2 TAP tag was bound to the IgG sepharose beads. The TAP tagged proteins enrichment to the IgG beads were revealed by Western blot analysis (Figure 1A-ii). The IgG bound proteins were eluted by digestion with AcTEV protease. The AcTEV cut eluate was incubated with CAM agarose beads. The protein enrichment by the CAM beads was detected by Western blot analysis (Figure 1A-iii). Finally, OsFIE2-TAP complex proteins were eluted with buffer containing EGTA. The eluate was TCA precipitated and processed for mass spectrometry analyses after trypsin digestion.

The complex proteins were identified using LC-MS/MS mass spectrometry analyses with the protein identification criteria of minimal two peptides as we reported previously [58]–[60]. The OsFIE2 complex includes OsFIE2 (homolog of ESC), OsCLF (homolog of E (Z)), OsSET1 (homolog of E (Z)), and *Os*EMF2b (homolog of Su (Z) 12) (Table 1). The histone H4 protein was identified in this study (Table 1). It is reported that *Drosophila* histone H4 strongly binds to polycomb protein p55 [61]. The RbAp48/46, mammalian polycomb protein, also binds to the histone H4 [62], [63]. Whether H4 is a stable component of the rice OsFIE2 complex remains to be further verified although it is possible. Two other proteins identified in our purification were elongation factor 1-alpha and heat shock cognate 70 kDa protein 2. These two proteins had been identified in an affinity purification using the same TAP system in rice and were considered as nonspecific interaction [53]. We also identified the heat shock proteins during the purification of another protein using the same TAP system (Mujahid and Peng, unpublished results). Therefore, we concluded that these two proteins were not the interactive proteins of the *OsFIE2-TAP* complex.

**Table 1:** 2D-LC-MS/MS analysis of OsFIE2 associated complex proteins identified by Tandem affinity purification.

Protein	Accession	Molecular Mass (Da)	P(Pro)	Coverage	Peptides
OsFIE2	LOC_Os08g04270	42035.7	1.62E-07	18.9	10
OsEMF2b	LOC_Os09g13630	68615.1	1.26E-05	6.10	2
OsCLF	LOC_Os06g16390	100295.3	5.44E-05	1.60	2
OsSET1	LOC_Os03g19480	99862.4	7.76E-05	1.20	1
Histone H4	LOC_Os01g61920	11409.3	9.37E-07	12.6	2

### OsFIE2 interacts with OsSET1 in *Escherichia coli* two-hybrid system

To understand the structure of the OsFIE2-PRC2 complex, we carried *E. coli* two-hybrid assays for pairwise interactions of the four polycomb subunits. *E. coli* two-hybrid assay has some advantages over yeast two-hybrid such as *E. coli* grows much faster than yeast and it can be transformed with higher efficiency so larger number of interactions can be rapidly screened. In addition, *E. coli* two-hybrid assay reduces the chance that the host harbors a eukaryotic homologue of one of the interacting protein partners [64]. Our results showed that OsFIE2 interacted with OsSET1 strongly (Figure 1A-iv). However, no other interactions were detected using the *E. coli* two-hybrid system. The results support that OsSET1 is a component of the OsFIE2 complex and suggest that post translational modification(s), other cellular component(s) such as long non-coding RNAs, or the nuclear environment is required to reveal other protein-protein interactions within the OsFIE2 complex.

### *In vitro* activity of TAP purified OsFIE2 complex

**Figure 2:**
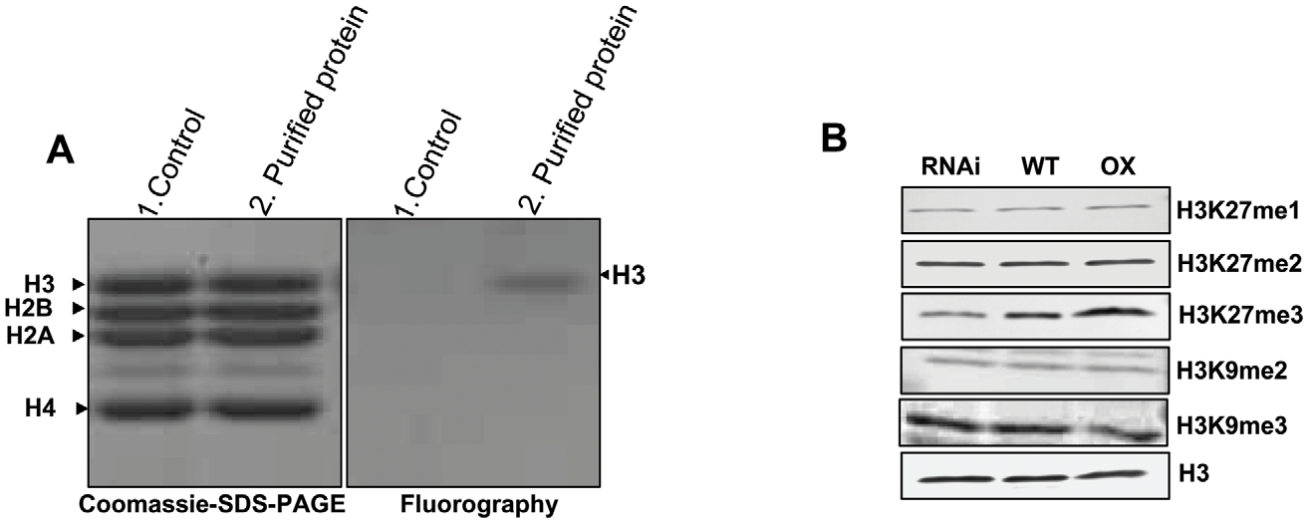
*In vitro* histone methyl transferase assay and immunoblot analyses of H3 modifications in transgenic lines. (A) *In vitro* histone methyltransferase assay of TAP purified OsFIE2-PRC2 complex: Chicken core histones were incubated with *S*-adenosyl-[methyl-^3^H]-l-methionine and affinity purified OsFIE2-PRC2 complex proteins. In the control reaction, TAP elution buffer was used instead of purified OsFIE2-PRC2 complex proteins. Methylated [^3^H] histones were resolved by 15% SDS-PAGE, stained with coomassie (Left side) and visualized by fluorography (Right side). (B) H3 methylation status in *OsFIE2*-RNAi line NO3, *OsFIE2*-overexpression line 04A and wild type plants. Equal amounts of protein samples were loaded for Western blot analyses. The specific antibodies used are indicated on the right and the protein sample sources are shown on the top. Western blot signals were normalized with the unmodified H3 band as control using the PDQUEST software.

Mammalian and *Drosophila* PRC2 complexes are shown to methylate H3K27 (histone H3 at lysine 27) and, to a lesser extent, H3K9 both *in vivo* and *in vitro* [65]–[68]. The substrates include H3K27, H3K27me, H3K27me2, and H3K9me. Although VRN complexes have been purified in *Arabidopsis*, no enzyme activities are reported *in vitro*. We carried out *in vitro* histone methyltransferase assay using the TAP purified OsFIE2 protein complex as enzyme, chicken core histones as substrate, and *S*-adenosyl-[methyl-^3^H]-l-methionine as methyl donor. The TAP purified OsFIE2-PRC2 complex demonstrated strong methyltransferase activity against histone H3 only (Figure 2A), indicating that our purified OsFIE2-PRC2 complex is a functional complex with H3 specific enzyme activity.

To further illustrate the enzyme specificity of the OsFIE2-PRC2 complex, we examined the H3K27 modification state in the *OsFIE2* overexpression line and the *OsFIE2* RNAi lines. Our results showed that H3K27me3 was reduced to about 54% in the RNAi line NO3 (Figure 2b). Meanwhile, H3K27me3 level had increased to about 152% in the overexpression line 04A compared with the wild-type. In contrast, the content of H3K27me and H3K27me2 had no detectable change, suggesting that *OsFIE2* PRC2 complex specifically catalyzed the conversion of H3K27me2 to H3K27me3. We also examined H3K9me2 and H3K9me3 levels in *OsFIE2* overexpression and RNAi lines (Figure 2B). The results indicated that H3K9 methylation state was not affected by *OsFIE2* overexpression or reduction, suggesting that H3K9me and H3K9me2 are not the primary substrates of the *OsFIE2*-PRC2 complex at genome level compared with H3K27me3. However, we can not rule out the possibility of the gene specific effect of OsFIE2 PRC on H3K9me and H3K9me2 in the rice genome. To further validate the results, we tested other RNAi and overexpression lines. Similar results and conclusions were obtained (Figure S1).

### Generation of *OsFIE2-*RNAi and overexpression transgenic rice plants

**Figure 3:**
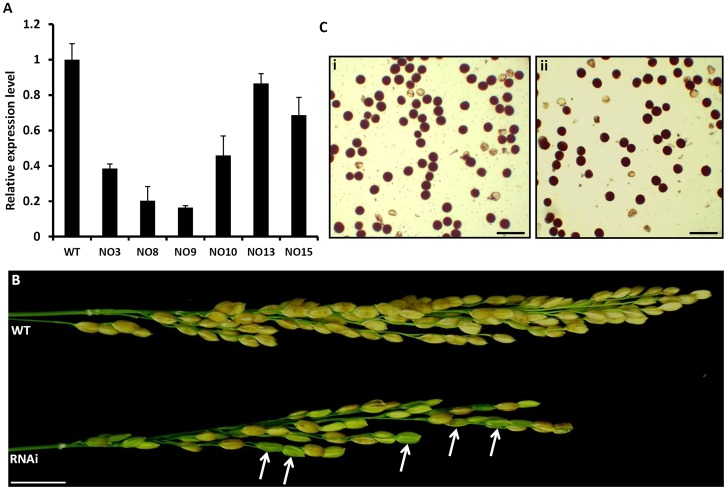
Analysis of *OsFIE2*-RNAi plants. (A) Quantitative real-time PCR analysis of *OsFIE2* gene expression in different RNAi lines and wild type. Rice Glyceraldehyde 3-phosphate dehydrogenase (*GAPDH*) was used as internal control. Same amount of cDNA template was used in each sample. Primers are listed in Table S3. (B) Comparison of mature panicles of *OsFIE2*-RNAi line NO3 (bottom) and wild type (top). Panicles of RNAi plants contain more partially filled seeds and less filled seeds when compared to wild type panicles. Partially filled or unfilled seeds are marked with arrow in panicle of OsFIE2-RNAi plant. (C) Images of pollen viability tests. Pollen grains are stained with I_2_-KI solution. Viable pollen grains are stained dark and nonviable pollen grains are stained light yellow. (i) Image of wild-type pollen. (ii) Image of *OsFIE2*-RNAi line NO3 pollen. The scale bars = 2 cm in (B) and 100 µm in (C).

To study the biological role of *OsFIE2* in rice, we made *OsFIE2*-RNAi constructs and obtained twenty independent RNAi lines using *Agrobacterium* mediated transformation. Six independent RNAi transgenic lines (No3, No8, No9, No10, No13 and No15) with different *OsFIE2* expression levels as revealed by quantitative real time PCR (Figure 3A) were selected for further analysis. No 8 and No 9 had less than 20% of the expression level of the wild-type. No 3 and No 10 had about 40% to 50% expression levels and No15 and No 13 had about 70% to 90% expression levels. We categorized Lines No8 and No9 as severe RNAi lines and the others as weak RNAi lines. The vegetative development of the weak RNAi plants was the same as the wildtype.

**Figure 4:**
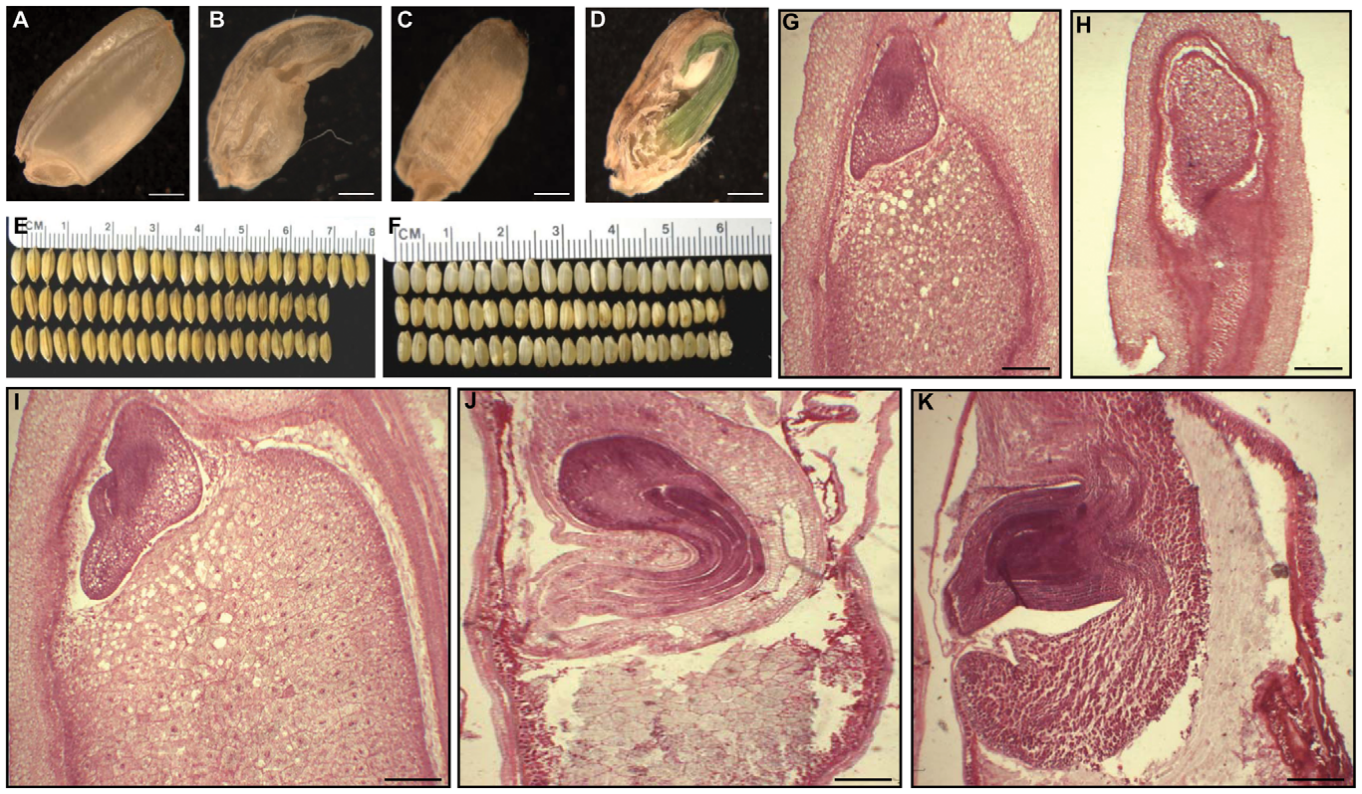
Phenotypes of *OsFIE2*-RNAi seeds. (A) Mature wild type seed with well-developed starchy endosperm. (B–D) Phenotype severity levels of partially filled seeds of *OsFIE2*-RNAi plant. (B) Severe partially filled seed phenotype of *OsFIE2*-RNAi plant with defective shrunken endosperm due to lack of proper starch filling. (C) Partially filled seed of *OsFIE2*-RNAi plants with partially filled shrunken endosperm. (D) Partially filled and lack of dormancy seed of the *OsFIE2*-RNAi plant. (E) Comparison of mature seeds of *OsFIE2*-RNAi line NO3 (bottom two rows) and wild-type (top row). (F) Comparison of mature seeds (without husk) of *OsFIE2*-RNAi line NO3 (bottom two rows) and wild-type (top row). Seed size of the *OsFIE2*-RNAi seeds is substantially smaller compared to the wild type seeds. (G,H) Images of longitudinal sections of *OsFIE2*-RNAi and wild-type early stage seeds (6 days After Fertilization). (G) Wild type early seed with well-developed starchy endosperm and viable embryo. (H) Image of partially filled early seed of *OsFIE2*-RNAi plants. Please compare the size ratio of embryo to seed in this seed with wild-type seeds (G). (I–K) Images of longitudinal sections of *OsFIE2*-RNAi and wild-type mature seeds. (I) Wild type mature seed with well-developed starchy endosperm and viable embryo. (J,K) Images of partially filled mature seeds of *OsFIE2*-RNAi plants with partially filled shrunken defective endosperm and large embryo. The scale bars = 1 mm in (A) to (D) and 100 µm in (G–K).

Phenotype evaluation of seed development in *OsFIE2*-RNAi T1, the endosperm was at T2 generation, and generations following revealed substantial differences compared with the wild-type. The ratio of mature and fully filled seeds was substantially reduced in RNAi lines (Figure 3B and Table 2). Fully filled mature seeds were produced in a ratio ranging from 66% to 81% in the weak RNAi lines, while the wild-type was about 88%. Interestingly, per thousand seed weight of the fully filled seeds in the weak RNAi lines was smaller in all the tested lines (Figure 4E, 4F and Table 2). The rest of the seeds were partially filled or not filled (Figure 4B). For the partially filled seeds, we found that some of them lost dormancy with seed germination before the seed mature (Figure 4D). We carried out a series of sectioning to examine the seed development in both wildtype and the RNAi lines. Defective shrunken endosperm tissue was observed in the partially filled seeds of RNAi lines (Figure 4H, 4J and K). In addition, we observed big embryos in the partially filled seeds without clear germination, which might be due to partial germination or uncontrolled embryo development (Figure 4J and 4K). Overall, the embryo sizes in the partially filled seeds increased to different degrees. Interestingly, the *Arabidopsis fie* mutants showed substantial delay in germination in contrast to loss of dormancy [34].

**Table 2:** Agronomic traits of T1 generation *OsFIE2*-RNAi transgenic lines.

Line Name	Partially filled or unfilled seeds (%)	Filled Seeds (%)	Seed Setting (%)	1000 seed Weight (g)	Plant Height (Cm)
NO3	21.70±0.82**	66.26±1.78**	87.98±1.61	19.53±0.30**	104.66±3.05*
NO8†	87.81±2.42**	0	87.81±2.42		99.0±1.0**
NO9†	88.90±1.42**	0	88.90±1.42		99.33±0.57**
NO10	19.30±1.79**	67.43±1.63**	86.79±0.26	20.03±0.20**	96.33±1.15**
NO13	7.61±2.60*	81.19±3.49	88.81±0.99	22.23±0.15**	105.0±4.35
NO15	10.0±0.93**	78.25±0.44**	88.26±1.09	22.16±0.25**	106.0±4.0
WT	1.68±1.0	88.05±0.86	89.74±1.60	25.33±0.15	113.66±1.52

* *P*<0.05 when compared with wild type (WT).

** *P*<0.01 when compared with wild type (WT).

† NO8 and NO9 *OsFIE2*-RNAi lines produced no viable filled seeds for progeny. The data is the T0 generation seeds instead of T1 generation.

The T_0_ plants of the two severe *OsFIE2*-RNAi lines (No8 and No9) did not produce viable seeds. We failed to obtain the reproductive progeny of the two transgenic lines although we successfully maintained the plants by asexually amplifying new tillers. To verify the phenotype observed in the severe lines, we carried out a second batch of transformation and generated another 18 independent lines. One line displayed severe phenotype but also failed to produce viable seeds. Since all the three severe RNAi lines failed to produce viable progeny, we could not verify if the observed phenotype is inheritable meiotically. Therefore, the phenotypes of the severe lines are not discussed further in this manuscript. The small seeds and partially filled seed phenotype were repeatable in the weak RNAi lines generated in the second batch (data not shown). To examine pollen quality, we carried out pollen viability test using iodine-potassium iodide staining method (Figure 3C). We found that the viable pollen ratio was about 87% in wild-type and about 86% in weak RNAi lines, suggesting that the pollen grains developed normally.

**Figure 5:**
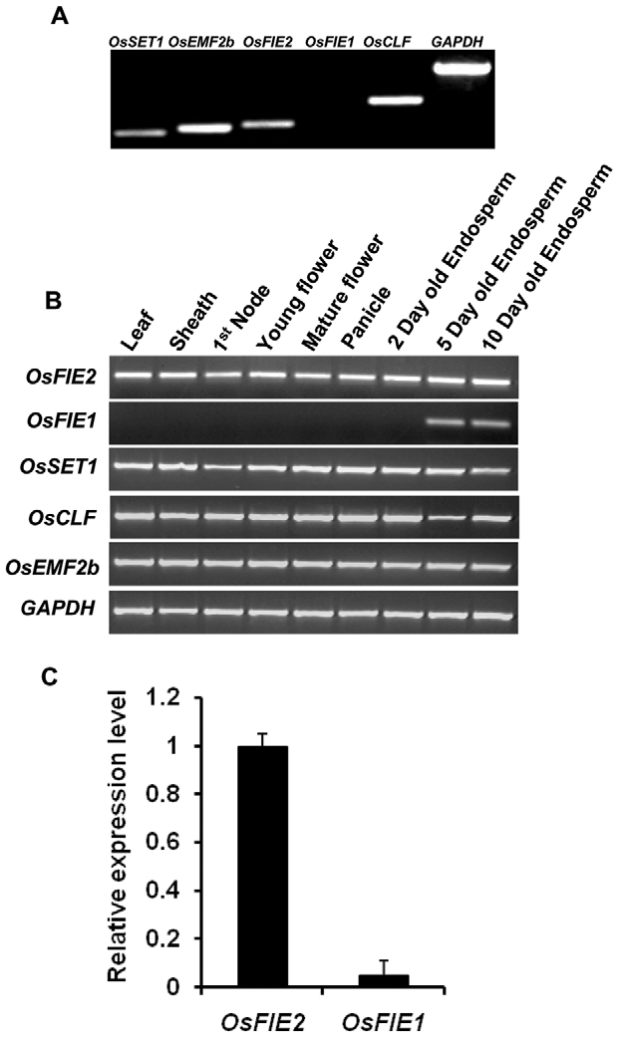
Expression profile analyses of polycomb genes in different rice tissues. GAPDH was used as internal control. Same amount of cDNA template was used in each sample. Primers are listed in Table S3. (A) Reverse Transcription-PCR analysis of the expression of the rice polycomb genes in anther tissue. 28 cycles were used. (B) Reverse-Transcription-PCR analysis of the expression of rice polycomb genes in different rice tissues. (C) Comparison of *OsFIE2* and *OsFIE1* expressions in 5-days old rice endosperm by quantitative real-time PCR.

Since autonomous endosperm development was reported in *Arabidopsis FIS/FIE* mutants, we emasculated anthers before anthesis in over 100 spikelets in the RNAi severe and weak lines, respectively. After examination with microscopy and the naked eye, no sign of autonomous endosperm development was observed in the absence of fertilization event. Meanwhile, we examined the expression of the *OsFIE2* gene and other polycomb group genes in anthers with reverse transcription PCR to study if these genes subject to imprinting regulation as in *Arabidopsis*. The *OsFIE2* gene was highly expressed while *OsFIE1* gene expression was not detected in anthers (Figure 5A). We also examined the expression of other *OsFIE2*-PRC2 complex genes and *OsFIE1* in other plant tissues, including leaf, sheath, stem nodes, young and mature flowers, panicles, and endosperm tissues in three different stages. All the *OsFIE2* complex genes were highly expressed in these tested tissues except that *OsCLF* might have minor quantity changes in five days old endosperm tissues (Figure 5B). In contrast, *OsFIE1* was only detected in five days and ten days old endosperm tissues. Luo et al. (2011) [45] carried out a genome wide analysis of imprinted genes in rice endosperm. They found that only *OsFIE1* but not other polycomb genes were imprinted in rice endosperm. Given that *OsFIE2* was highly expressed in anther but not *OsFIE1*, our results were consistent with their report (Luo et al. 2011) [45] that *OsFIE1* was probably paternal imprinted but not *OsFIE2*. The dominant negative nature of RNAi line prevented us to further test if *OsFIE2* is imprinted by reciprocal crosses.

Surprisingly, the endosperm specific *OsFIE1* mutant had no endosperm phenotype [42], but the endosperm unspecific gene *OsFIE2* RNAi lines all had reduced seed size and partially filled phenotype. We compared *OsFIE1* and *OsFIE2* expression level in endosperm with real-time PCR (Figure 5C). The results showed that the *OsFIE1* expression level was only about 4% of the expression level of *OsFIE2* in endosperm. The *OsFIE1* and *OsFIE2* proteins share 72% of overall amino acid sequence identities. If the N-terminal 82 amino acid stretch specific for *OsFIE1* is excluded, the two proteins share about 85% amino acid sequence identities. If *OsFIE1* and *OsFIE2* are functionally redundant, our results would imply that *OsFIE1* level is too low to replace the function of *OsFIE2* because a reduction of *OsFIE2* expression to 20% could not produce viable progeny but the *OsFIE1* expression is only 4% of the *OsFIE2*gene. In contrast, *OsFIE2* level is high enough to replace *OsFIE1* function. Therefore, mutation on *OsFIE1* gene may not show phenotype, but mutation on the *OsFIE2* gene will show phenotype. Due to the high homology between *OsFIE2* and *OsFIE1*, we tested if the *OsFIE1* expression was also reduced in the *OsFIE2* RNAi lines. Our results showed that *OsFIE1* expression was not affected in the *OsFIE2* RNAi lines (Figure S3).

We also generated multiple *OsFIE2* overexpression transgenic lines. We found no obvious phenotype in all the transgenic plants and the endosperm and embryo development were normal as wild type (data not shown).

### *OsFIE2* regulates the production of storage proteins and storage starch in the rice endosperm

**Figure 6:**
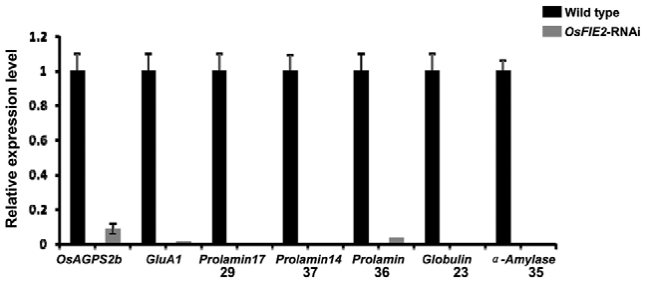
Quantitative real-time PCR analyses of gene expression in the storage protein and starch synthesis pathways in the *OsFIE2* RNAi and wild-type endosperm. We used GAPDH as internal control and same amount of cDNA template was used in each sample. Numbers 29, 37, 36, 23, 35 indicates the genes encoding proteins corresponding to 2-DE gel spot numbers shown in Figure 7 and Table 3.

Given that the mature seeds of the RNAi lines are much smaller than the wild-type and many seeds are only partially filled, we examined the expression levels of selected starch biosynthesis pathway genes in the endosperm using quantitative real-time PCR. The healthy looking developing seeds of weak RNAi lines in the grain filling stage were selected for the experiment. The genes tested included ADP-glucose pyrophosphorylase small subunit 1 (AGPS1)-AK073146, AGPS2a-AK071826, AGPS2b-AK103906, AGPL1-AK100910, AGPL2-AK071497 and AGPL3-AK069296. Among them, the expression of *AGPS2b* reduced to 9% compared with the wild type (Figure 6). Other genes had no substantial change. Our results indicated that the *AGPS2b* gene, encoding the rate limiting step enzyme in the starch biosynthesis pathway [69] is subjected to the regulation of *OsFIE2* either directly or indirectly.

**Figure 7:**
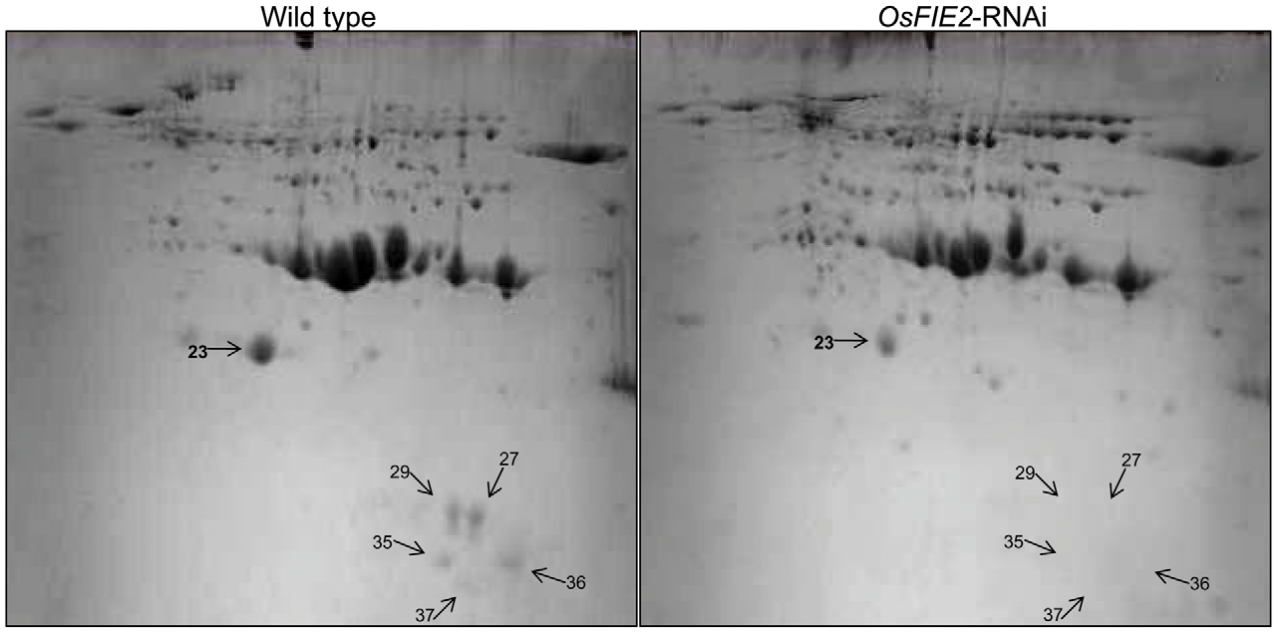
2-DE gel images of rice seed proteins from weak *OsFIE2*-RNAi and wild-type plants. Proteins were extracted from fully filled mature rice seeds, separated by 2-DE , and visualized by coomassie blue staining. Differentially expressed protein spots were marked with arrows and numbers.

During the seed filling stage, massive amounts of storage proteins are accumulated in cereal endosperm [70], [71]. To investigate the possible role of *OsFIE2* in the regulation of storage protein accumulation, we examined the storage protein content in the endosperm of fully filled mature seeds in the weak RNAi lines NO 3, NO 10, and NO15 using two-dimensional (2-DE) gel analysis. We isolated total proteins from the mature seeds of both *OsFIE2*-RNAi and wild-type seeds using phenol extraction method as we reported [72]. After 2-DE gel separation and PDQUEST analysis, differentially regulated protein spots were robotically excised and analyzed using MALDI TOF-TOF mass spectrometry. Proteins with reduced accumulation in the RNAi seeds included prolamin, globulin, alpha-amylase and seed allergenic proteins (Figure 7 and Table 3). The results were similar in all three tested RNAi lines. Real-time PCR analysis demonstrated the expression of some genes also reduced at the mRNA level (Figure 6), suggesting a regulation of these genes at the transcriptional level. We also found that the expression of *Glutelin A1* (Os01g55690) reduced in RNAi seeds compared with the wild type (Figure 6).

**Table 3:** Differentially regulated proteins identified in 2D-gel images of *OsFIE2* RNAi and wild-type seed proteins.

Spot No	Protein	Exp. MW (Da)	Exp. pI	Peptides	C.I. %	Locus No.
23	Globulin	21040.9	7.48	10	100	LOC_Os05g41970
27	Seed allergenic RAG2	19424.7	8.91	6	100	LOC_Os07g11410
	Seed allergenic RA5	17267.4	8.36	7	100	LOC_Os07g11510
	Seed allergenic RAG2	20494.2	9.01	6	99.6	LOC_Os07g11380
29	Prolamin 17	16663.7	8.25	6	100	LOC_Os06g31070
35	Alpha-amylase	16466	7.48	9	100	LOC_Os07g11650
	Alpha-amylase	16394.9	7.48	9	100	LOC_Os07g11630
	Retrotransposan Ty3	186292.1	8.42	24	99.6	LOC_Os11g12100
36	Prolamin	17794	8.66	3	100	LOC_Os07g10570
	Prolamin	17638.1	8.68	3	100	LOC_Os07g10580
	Seed allergenic RA5	17267.4	8.36	6	100	LOC_Os07g11510
37	Prolamin 14	16858.8	8.79	4	100	LOC_Os05g26377
	Prolamin 14	16951.8	9.26	3	100	LOC_Os05g26440
	Prolamin 14	16867.8	8.79	4	100	LOC_Os05g26350

### Genome-wide identification of H3K27me3 binding sites

Our studies above indicated that *OsFIE2* played a critical role in regulating endosperm development and grain filling and the *OsFIE2*-PRC2 complex specifically catalyzes the methylation of H3K27me2 to H3K27me3. We hypothesized that some key regulatory genes controlling storage starch and protein synthesis in endosperm must be subjected to the regulation of H3K27me3 either directly or indirectly. We have carried out a genome wide ChIP-Seq experiment using 6 to 7 days post pollination endosperm tissues as materials to compare four algorithms used for ChIP-Seq data analysis [73]. The endosperm ChIP-Seq results were verified by ChIP-PCR using 28 selected genes [73] and the antibody used for the experiment (antibodies against H3K27me3, produced by Millipore) is the antibody with 100% specificity for H3K27me3 [74]. Given that the role of H3K27me3 is primary for gene repression and 6 to 7 days post pollination is in the stage before starch and storage protein synthesis, we expected that H3K27me3 should be still associated with genes regulating storage nutrient synthesis in this stage. Therefore, we decided to use the published data set to search for genes regulated by H3K27me3 modification in endosperm. Given that the antibody might bind to non specific sites in the mutant background due to reduced H3K27me3 [34], the results obtained in wild-type background should be the best one could get.

Malone et al. (2011) [73] compared four Algorithms for ChIP-Seq data analysis and concluded that the overall biological conclusion of the results obtained by the four algorithms is the same. Since the FindPeak algorithm combines excellent sensitivity with accuracy compared with the other three Algorithms [73], the findPeak results are discussed here. The FindPeak program analyses found that 76 endosperm specific genes, including DNA binding proteins, and 97 nutrition metabolic pathway genes were enriched by antibodies against H3K27me3 in the endosperm ChIP-Seq experiment (Table S1). The total list of enriched peaks is shown in Table S2. The selected examples of the peak profiles are shown in Figure S2. Examples of enriched endosperm specific genes include: MYB protein, putative (LOC_Os02g09670); helix-loop-helix DNA-binding domain containing protein (LOC_Os04g35010); basic region leucine zipper domain containing protein (LOC_Os09g34880); RNA recognition motif containing protein (LOC_Os09g14550); trehalose-6-phosphate synthase (LOC_Os09g25890); OsIAA29-Auxin-responsive Aux/IAA gene family member (LOC_Os11g11430); etc. The ChIP enriched nutrition metabolic pathway genes include: seed specific protein Bn15D1B (LOC_Os04g50970); starch synthase (LOC_Os06g04200); globulin 2 (LOC_Os11g34780); glutelin (LOC_Os02g15090); seed maturation protein PM23 (LOC_Os03g41080); starch binding domain containing protein (LOC_Os05g37450); etc.

### *OsFIE2* regulates the histone modification and expression of a helix-loop-helix DNA–binding gene

**Figure 8:**
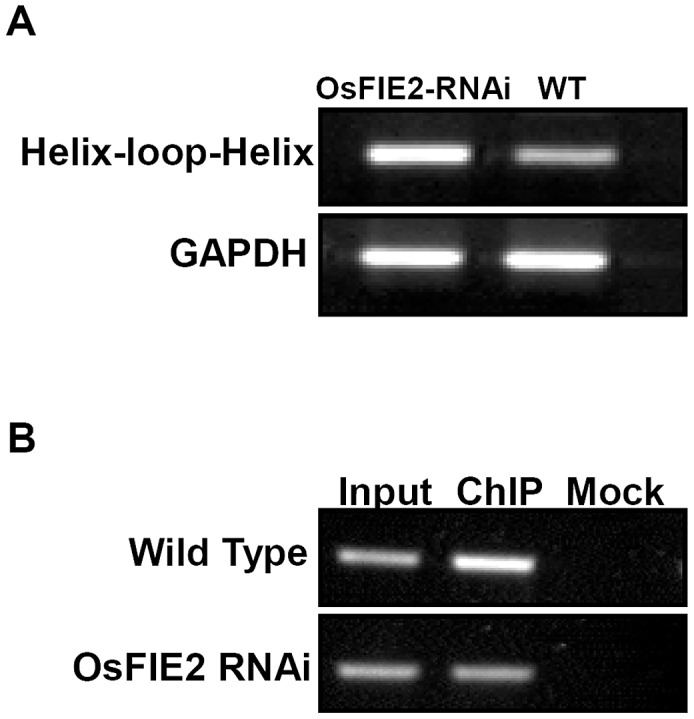
Reverse Transcription–PCR and ChIP PCR analysis of helix-loop-helix DNA binding domain containing gene in wild-type and *OsFIE2*-RNAi plants. (A) Reverse Transcription-PCR analysis of the expression of helix-loop-helix DNA binding domain containing gene in endosperm tissue. GAPDH was used as internal control. Same amount of cDNA template was used in each sample. Primers are listed in Table S3. (B) ChIP PCR analysis of helix-loop-helix DNA binding domain containing gene in wild type and *OsFIE2*-RNAi plants. Chromatin is isolated from endosperm tissue of wild type and *OsFIE2*-RNAi plants. Antibodies for H3K27me3 were used for chromatin immunoprecipitation (ChIP). Input: DNA sample extracted from the chromatin before ChIP enrichment. ChIP: DNA sample extracted from H3K27me3 ChIP enriched chromatin. Mock: DNA sample that went through the immunoprecipitation procedure without antibody.

Our ChIP-Seq results showed that the helix-loop-helix DNA-binding domain containing protein (LOC_Os04g35010) is enriched in the ChIP-Seq experiment for H3K27me3 modification (Figure S2 and Table S1). The gene's *Arabidopsis* ortholog RGE1 (AT1G49770) is expressed in the endosperm surrounding region which directly surrounds the developing embryo. It exerts its effect non autonomously in the developing embryo [75], [76]. Loss of RGE1 function results in small embryos. Mutant seedlings are extremely sensitive to desiccation due to the abnormal cuticle. Since we observed large embryo phenotype in our RNAi lines, we checked the expression of the rice *LOC_Os4g35010* gene in both wild-type and the RNAi lines in developing seeds. We found that this gene was over expressed in the RNAi lines compared with wild-type as shown in Figure 8. Further ChIP-PCR experiment using antibodies for H3K27me3 showed that the H3K27me3 modification was substantially enriched in this locus in the wild-type but the enrichment substantially reduced in the RNAi lines. Our results confirmed the ChIP-Seq result and suggest that this gene's H3K27me3 modification level and expression are regulated by *OsFIE2* expression.

## Discussion

### OsFIE*2* forms a multi-protein complex specifically catalyzing the production of H3K27me3

The role of polycomb group genes in plant development has been extensively studied in *Arabidopsis*. The polycomb complex that regulates vernalization process has been purified in *Arabidopsis* using Tandem affinity tag approach and the seven putative subunits of the complex have been identified following LC-MS/MS analysis [3], [14]. However, no enzyme activities had been tested *in vitro* for the PRC2 complexes in plants. Thus, the mechanism of PRC2 complex action has not been demonstrated in plants. Given the unique and essential function of PRC2 complexes in cereal crops, purifying and characterizing the complex in cereals is particularly important. In this report, we purified the OsFIE2-PRC2 protein complex in rice and demonstrated the Histone methyltransferase activity *in vitro*.

We find that OsFIE2 (the ESC homologue) forms a stable protein complex with polycomb proteins OsCLF (the E (Z) homologue), OsSET1 (the E (Z) homologue), and OsEMF2b (the Su (Z) 12 homologue). Our *E. coli* two hybrid studies show that OsFIE2 may directly interact with OsSET1. The *OsFIE2*-PRC2 complex genes are well expressed in young and mature endosperm tissues in rice. Along with the PRC2 core components, the histone H4 protein was co-purified with the OsFIE2 protein. Interaction of histone H4 with polycomb proteins was reported in mammals and *Drosophila*. Mammalian ortholog of p55, RbAp 48/46, directly binds to histone H4 [62], [63]. GST pull-down assay revealed that *Drosophila* histone H4 strongly and specifically binds to p55 [61]. Therefore, H4 may directly interact with *OsFIE2*-PRC2 complex although further experiments are required to confirm the interaction. There are four p55 homologous genes in the rice genome [46]. But we did not detect the p55 homologous proteins in purified complex although we successfully detected methyltransferase activity with the purified complex. One possibility is that the quantity of p55 homologous protein is very low in the complex. The mass spectrometer failed to detect it. Alternatively, the polycomb complex in rice has components different from those in other organisms. Nevertheless, it is clear that the rice OsFIE2-PRC2 complex is conserved in overall structure compared with the *Drosophila* PRC2 complex. Our *E. coli* two hybrid studies show that *OsFIE2* may directly interact with *OsSET1*. Other interactions were not detected in *E. coli* two hybrid assays among the components, suggesting that posttranslational modifications or additional components may be involved in complex formation. It is interesting to note that non coding RNAs play a key role in the polycomb complex [77], [78]. But it is still unknown if the RNAs play any critical structure role in the complex. Our expression studies show that all the *OsFIE2*-PRC2 complex genes are well expressed in both young and mature endosperm tissues of rice.

In Mammals and *Drosophila*, PRC2 complexes were shown to methylate histone H3 at lysine 27 and, to a lesser extent, H3K9 both *in vivo* and *in vitro* [65]–[68]. In *Arabidopsis* vernalization mutant *vrn2*, encoding a subunit of PRC2 complex, both methylation marks at H3K9 and H3K27 were lost [22], [79]. In two other mutants *vrn1* and *vin3*, the H3K9me2 mark is missing. Vin3 has also been shown to be a component of the PRC2 complex with VRN2 [3], suggesting a role of the PRC2 complex in regulating H3K9 methylation. Therefore, detecting the methyltransferase activity and revealing the substrate specificity of the *OsFIE2*-PRC2 complex is important for understanding the molecular mechanism of PRC2 complex function in plants. Our studies with the overexpression and RNAi lines demonstrated that manipulation of the *OsFIE2* gene expression level had no effect on the cellular level of H3K9me2, H3K9me3, H3K27me and H3K27me2 but resulted in a substantial change in the cellular level of H3K27me3, suggesting that OsFIE2-PRC2 complex is primary for regulating the formation of H3K27me3 at genome level. However, we can not rule out the possibility of the gene specific effect of OsFIE2 PRC on other modifications in the rice genome. The results suggest that the function of the rice OsFIE2-PRC2 complex is different from the *Arabidopsis* VRN2-PRC2 complex, the later contained more subunits and may have H3K9 methyltransferase activity [3], [22], [79]. The OsFIE2-PRC2 complex is also different from the *Arabidopsis* endosperm development related MEA-FIE complex because the orthologs of the *Arabidopsis MEDEA* and *FIS2* were not present in the rice genome.

### *OsFIE2* regulation model in rice seed and endosperm development is different from that in *Arabidopsis*

The polycomb gene *FIE* is well studied in *Arabidopsis* and is required to repress the endosperm development in the absence of fertilization [6], [7], [13], [15], [16]. Loss-of-function mutations of genes in the MEA-FIS- FIE-MSI1 complex can form autonomous diploid endosperm in the absence of fertilization [6], [7]. Mutation in *MSI1* gene exhibits very strong phenotype of autonomous endosperm development [8], [26]. It is shown that *MEDEA* represses expression of *Arabidopsis* MADS-box gene *PHERES1* during the seed development [8], [9]. Interestingly, *MEA* gene itself is one of the target genes of the FIS complex, indicating a self-imprinting mechanism in *Arabidopsis* [32], [33], [80].

We found that emasculated *OsFIE2*-RNAi florets did not show autonomous endosperm development. Similarly, no autonomous endosperm development was observed in rice T-DNA mutants of *OsFIE1* and *OsEMF2b* [42]. The maize RNAi plants of *ZmFIE1* and *ZmFIE2* also produced no autonomous endosperm in the absence of fertilization [46]. Further, RNAi lines of *HFIE* in sexual *Hieracium pilosella*, failed to show the autonomous central cell proliferation [48]. These results, together, suggested that the function of *FIE* gene is not to suppress endosperm development. The function of the FIE containing PRC2 complex in repressing autonomous endosperm development in *Arabidopsis* is not conserved among plants.

We found that down regulation of *OsFIE2* gene in RNAi plants resulted in the production of small seeds and partially filled seeds (Figure 3 and Figure 4). Our results suggested that the *OsFIE2* positively regulated endosperm development in rice instead of repressing endosperm development as predicted using *Arabidopsis* as the model. We found that some of the partially filled seeds germinated before maturation. Cross sectioning of the un-germinated partially filled seeds found that the embryos were bigger than the wild-type. It is still unknown if the large embryo was due to partial germination or other developmental defect. In addition, it is also unknown if the early germination is directly regulated by polycomb complex or it is a secondary effect due to partial filling or others.

Interestingly, the *Arabidopsis FIE* mutant showed delayed seed germination [34]. While the wild-type seeds germinated within 2 days, approximately 40% of the homozygous *FIE* mutants stayed dormant for the course of the entire experiment, which lasted 20 days. Therefore, the *FIE* containing polycomb complex in rice and *Arabidopsis* have distinct roles. While the *Arabidopsis* complex suppress endosperm development in the early stage and promote seed germination after maturation, the rice OsFIE2 complex, in contrast, is required for seed and endosperm development and suppress early germination of the seeds either directly or indirectly.

Interestingly, no phenotype was reported in mutants of the endosperm specific gene *OsFIE1*, a gene sharing the highest homology with *OsFIE2* in the rice genome. Given the high homology of these two genes, these two genes might be functionally redundant. Analysis of the gene expression profile indicates that the expression level of *OsFIE2* is about 25 times higher than that of the *OsFIE1* gene in the endosperm. If these two genes are truly redundant in function, *OsFIE1* mutant will display no phenotype because *OsFIE2* can replace *OsFIE1* function. In contrast, *OsFIE2* mutant will display phenotype because the expression level of *OsFIE1* is too low to replace the function of *OsFIE2*. Indeed, no phenotype was reported for *OsFIE1* mutant but striking phenotypes were observed in our *OsFIE2* RNAi lines.

### *OsFIE2* regulation in gene expression in seeds

Our real-time PCR results showed that the starch synthesis rate limiting step enzyme (glucose pyrophosphorylase) subunit *OsAGPS2b* was down regulated in the *OsFIE2* RNAi lines. Meanwhile, proteomics analysis revealed that the expression of prolamin, globulin, alpha-amylase, and seed allergenic proteins are reduced in *OsFIE2* RNAi lines (Figure 6, Figure 7, and Table 3). These results suggest that the partially filled and smaller seeds were probably due to a reduction of starch and storage protein synthesis.

To understand how the polycomb group genes regulate gene expression in rice endosperm, we analyzed our prior published rice endosperm ChIP-Seq data set. The ChIP-Seq data has been well verified by ChIP-PCR experiments with twenty eight genes [73]. We found that a large number of endosperm specific genes and storage nutrient metabolic pathway genes were subjected to the regulation of H3K27me3 in endosperm (Table S1 and Table S2). However, most genes shown to be down regulated by real-time PCR in the *OsFIE2* RNAi lines (Figure 6) are not the direct targets of the H3K27me3, suggesting a complicated gene expression regulation network in endosperm. Seed development is a complex quantitative trait. If the starch and protein synthesis genes were directly regulated by polycomb complex, it would act as a simple Mendelian trait. Therefore, it is understandable that many of the starch and storage protein genes are not the direct targets of OsFIE2 complex. In addition, there are multiple polycomb complexes in plants. It is also possible that many ChIP-Seq identified loci are not the direct target of *OsFIE2* complex but the targets of other PRC2 complexes.

*Arabidopsis* helix-loop-helix DNA binding domain containing gene *RGE1* (AT1G49770) is specifically expressed in the endosperm surrounding region which directly surrounds the developing embryo. It exerts its effect non autonomously in the developing embryo. Loss of RGE1 function results in small embryos. We found that the expression of rice *RGE1* gene was increased and the modification of H3K27me3 at the locus was reduced in the RNAi lines, suggesting that it is a potential target subject to *OsFIE2* regulation. Interestingly, while a mutation of the *RGE1* gene leads to small embryo in *Arabidopsis*, we observed large embryo in the RNAi lines showing high expression of the rice *RGE1* gene.

The MADS box gene *OsMADS6* (LOC_Os02g45770) is highly expressed both in flower and endosperm. Prior studies have shown that *OsMADS6* is subjected to the regulation of H3K27me3 [71] and our ChIP-seq data analysis results verified that *OsMADS6* was enriched by antibodies for H3K27me3 (Table S1 and S2). *OsMADS6* controls grain filling by regulating the gene encoding OsAGPS1 (ADP-glucose pyrophosphorylase small subunit1, AK073146), a subunit of the ADP-glucose pyrophosphorylase [71]. Meanwhile, we found that the expression of another subunit gene *OsAGPS2b* of the glucose pyrophosphorylase enzyme was down regulated in the *OsFIE2* RNAi lines. Further, a starch synthase and several storage protein genes were shown to be enriched in the ChIP-Seq experiment. Our results suggest that the *OsFIE2* polycomb complex controls endosperm development and grain filling by regulating multiple levels of targets including transcription regulators as well as metabolic pathway genes. The large number of genes subject to the regulation of H3K27me3 in endosperm is consistent with the critical role of *OsFIE2* gene in endosperm development and grain filling and suggests a highly sophisticated regulatory network.

## Materials and Methods

### Plant materials and growth conditions

Rice (*Oryza sativa*, cultivar Nipponbare) growth conditions were similar to our previous report [81]. All rice plants were grown in the greenhouse of the Department of Biochemistry and Molecular Biology, Mississippi State University, MS, USA. Wild-type Nipponbare (rice subspecies japonica) was used as control.

### Generation of TAP-tagged *OsFIE2* overexpression transgenic plants and cell suspension cultures

The *OsFIE2* full length cDNA clone (AK111761, Jo23058f21) was obtained from National Institute of Agrobiological Sciences, Ibaraki, Japan. The cDNA was amplified using primers 61TAPF and 61TAPR and cloned into pENTR™/D-TOPO (Invitrogen) entry vector. The cDNA clone in the entry vector was transferred to final UbI-NTAP-1300 destination vector [51] by using a single LR clonase recombination reaction (Invitrogen). The final N-Terminal TAP-tagged *OsFIE2* construct was transformed to *Agrobacterium* strain EH105A by electroporation. Rice transformation was performed as described Wu et al. (2003) [82]. Transformed resistant callus was screened using hygromycin. Transgenic plants were regenerated from the resistant callus. Mature seeds from the T1 transgenic plants with high expression of TAP-tagged *OsFIE2* were used to induce transgenic callus. Cell suspension cultures were generated from transgenic callus and maintained in suspension medium (3.2 g/liter Gamborg B5 basal medium (Phytotechnology Laboratories™), 0.5 g/liter MES, 20 g/liter sucrose, 2 mg/liter 2, 4-Dichloro acetic acid, 2 g/liter N-Z-AmineA, P^H^ 5.7, adjusted with 1 M KOH) at 25°C in darkness by constant shaking (150 rpm) on a gyratory shaker.

### Generation of *OsFIE2*-RNAi transgenic lines

A portion of coding sequence fragment was amplified using primer set 61RNAiF and 61RNAiR from *OsFIE2* cDNA clone (AK111761, Jo23058f21) and cloned into pENTR™/D-TOPO (Invitrogen) entry vector. The amplified fragment in the entry vector was transferred to final pANDA vector [83] by using a single LR clonase recombination reaction (Invitrogen). The final *OsFIE2*-RNAi construct was transformed to *Agrobacterium* strain EH105A by electroporation. Rice transformation was performed as described Wu et al. (2003) [82]. Transformed resistant callus was screened by using hygromycin. Transgenic plants were regenerated from the resistant callus.

### Tandem affinity purification

Exponentially growing cell suspension cultures (30 g) were harvested 3 days after subculturing and ground in liquid nitrogen. Protein extracts were prepared in two volumes of extraction buffer (20 mM Tris-Hcl, pH 8.0, 150 mM NaCl, 0.1% IGEPAL (Sigma), 2.5 mM EDTA, 2 mM benzamidine, 10 mM β-mercaptoethanol, 20 mM NaF, 2 mM phenyl methanesulfonylfluoride (PMSF), 1% Protease cocktail (Sigma), 10 µM leupeptin (Sigma), and 10 µM 3,4-dichloroisocoumarin (Sigma)). The suspension was homogenized with the help of Polytron PTA 20 TS homogenizer for 2 min on ice and filtered through two layers of miracloth. The soluble protein fraction was collected by centrifugation twice at 30,000 g for 20 min at 4°C. Affinity purifications were performed as described by Rohila et al. (2006) and Van Leene et al. (2007) [53], [54] with some modifications. The protein extract was incubated with 400 µl IgG sepharose beads (GE Healthcare) for 1 h at 4°C. The IgG beads were loaded on to a polyprep chromatography column (Bio-Rad Laboratories, CA, USA) and washed with 10 ml of extraction buffer lacking protease inhibitors and 10 ml of TEV (Tobacco etch virus) cleavage reaction buffer (10 mM Tris–HCl, pH 8.0, 150 mM NaCl, 0.1% IGEPAL, 0.5 mM EDTA, 1 mM DTT). Bound proteins were eluted by digestion with 150 U of Ac TEV enzyme in TEV cleavage reaction buffer containing 1 µM E-64 protease inhibitor for 1 h at 16°C. IgG-eluted fraction was adjusted to 2 mM Cacl_2_ and diluted with 3 volumes of calmodulin binding buffer (10 mM Tris–HCl pH 8.0, 150 mM NaCl, 1 mM Mg-acetate, 1 mM imidazole, 2 mM CaCl_2_, 0.1% IGEPAL, 10 mM *β*-mercaptoethanol) and the fraction was incubated with 400 µl of calmodulin-agarose beads (Stratagene, CA) for 1 h at 4°C. The calmodulin-agarose beads were loaded on to a polyprep chromatography column and washed with 10 ml of calmodulin binding buffer. The protein complexes were eluted with 2 ml of elution buffer (10 mM Tris–HCl pH 8.0, 150 mM NaCl, 1 mM Mg-acetate, 1 mM imidazole, 25 mM EGTA, 0.1% IGEPAL, 10 mM *β*-mercaptoethanol) and proteins were precipitated with TCA. The protein pellet was washed with cold acetone.

### Mass spectrometric analysis of TAP purified complex proteins

Tandem affinity purified complex proteins were trypsin digested and identified using LC-MS/MS mass spectrometry analysis as described by Chitteti et al. (2008); Tan et al. (2007); Tan et al. (2010) [58], [59], [60].

### Western blot analysis

Protein extracts were prepared according to the TAP protocol. Protein samples were collected in different stages of the TAP purification and separated on a 12% SDS-polyacrylamide gel and transferred on to PVDF (Millipore) membrane. In the first step of purification, presence of TAP-tagged protein was identified by using peroxidase anti peroxidase conjugate (PAP, Sigma) antibody, which is specific to protein A domain of the TAP tag, as previously described Rivas et al. (2002) [56]. In the second step of purification, CBP domain was detected by using biotinylated CAM as previously described Rohila et al. (2004) [51].

To check the methylation status, protein extracts from *OsFIE2*-RNAi, *OsFIE2*-overexpression and wild type leaf tissues were separated by 15% SDS-PAGE gel and transferred to immobilon membrane. Immunoblots were performed using antibodies for H3K27me1, H3K27me2, H3K27me3, H3K9me2, H3K9me3 and unmodified H3 (Table S4) by following the standard Western blot procedure [59]. Equal amounts of protein samples were loaded for Western blot analysis. Western blot signals were normalized using unmodified H3 band intensity as a control and quantified with the help of PDQUEST software.

### *In vitro* histone methyltransferase assay

Procedures for histone methyltranferase assay were adapted from the reported method Li et al. (2002) [84]. Briefly the assay was carried out by incubating TAP purified *OsFIE2* protein complex, 10 µg of chicken core histones (Upstate) and 2 µCi of *S*-adenosyl-[methyl-^3^H]-l-methionine (GE Healthcare) in 50 µl of reaction buffer (50 mM Tris–HCl, pH 8.5, 20 mM KCl, 10 mM MgCl_2_, 1 mM CaCl_2_, 10 mM 2-mercaptoethanol, 1 mM dithiothreitol, and 250 mM sucrose) for 2 h at 30°C. The reaction was stopped by addition of SDS loading buffer and boiling at 100°C for 10 minutes. The proteins were separated by 15% SDS-PAGE gel and visualized by coomassie staining and fluorography.

### Protein extraction and two-dimensional polyacrylamide gel electrophoresis

Total seed proteins were isolated from the mature seeds of both *OsFIE2*-RNAi and wild type plants. Mature seeds were ground into fine powder and protein extractions were performed using phenol extraction method as described Chitteti and Peng (2007) and Li et al. (2008) [72], [85]. Eight hundred micrograms of seed proteins were separated by using a standard 2-DE gel electrophoresis as described by Chitteti and Peng (2007) [85]. After PDQUEST analysis, the spots of interest were robotically excised from 2-DE gels by proteome works spot cutter (BioRad). In-gel digestion and MALD TOF-TOF mass spectrometry was performed as described by Chitteti and Peng (2007) [85].

### RNA isolation and RT–PCR analysis

Total RNA extraction from different tissues of rice plants was performed by using Trizol (Invitrogen) according to the manufacturer's instructions. Three micrograms of total RNA was used for the cDNA synthesis by using Moloney murine leukemia virus (M-MLV) Reverse Transcriptase (Invitrogen). Rice Glyceraldehyde 3-phosphate dehydrogenase (*GAPDH*) (Loc_Os04g40950) was used as an internal control. PCR products were analyzed by 1% agarose gel electrophoresis. Primers used in the experiment are listed in Table S3.

### Microscopy

Rice developing grains were fixed in FAA (3.7% formaldehyde, 5% acetic acid, 50% ethanol) at 4°C for overnight. Samples were dehydrated through graded ethanol series. After infiltration with xylene, samples were embedded in paraffin (Sigma-Aldrich), sectioned at 10 µm, and stained with 0.1% Eosin Y. Sections were observed and photographed using a bright-field microscope (Nikon).

### Quantitative real-time PCR analysis

Real-time quantitative PCR analysis was performed with LightCycler 480 Real-Time PCR System (Roche Applied Science), using LightCycler 480 SYBR Green I Master. The relative quantification of transcript levels was performed by using 2^−ΔΔ*C* T^ Method [86]. The rice GAPDH (Loc_Os04g40950) gene was used as an internal control. Primers used in the experiment are listed in the Table S3.

### *E. coli* two-hybrid assay

*E. coli* two hybrid assay was carried out according to the instructions given by manufacturers (Agilent Technologies). Recombinant bait (pBT) and target (pTRG) vectors were co-transformed into BacterioMatch II competent cells and positive interactions were screened by using selective and dual selective medium [64].

### Pollen viability assay

To examine the pollen viability of *OsFIE2*-RNAi and wild-type plants, anthers were collected from well developed spikelets just before anthesis. Mature pollen grains were stained with 1% iodine in 3% potassium iodide solution (KI-I_2_) [87].Viable pollen grains were stained black and nonviable pollen grains were stained light yellow. The viable pollen grains were examined and counted under microscope.

### Chromatin extraction and chromatin immunoprecipitation

The chromatin was extracted from endosperm following the protocol of Gendrel et al. (2005) [88] with minor modifications as reported recently Malone et al. (2011) [73]. Chromatin immunoprecipitation (ChIP) experiments were performed as reported Malone et al. (2011); Gendrel et al. (2005) [73], [88] using H3K27me3 antibody (Millipore) and protein A agarose/Salmon Sperm DNA.

### Library preparation and solexa sequencing

The library preparation and Solexa Sequencing were the same as reported Malone et al. (2011) [73]. Input and ChIP samples were processed following Illumina's protocol from the ChIP DNA Sample Prep Kit. Briefly, 10 ng input and ChIP enriched DNA was subjected to end repair, addition of “A” bases to 3′ ends, ligation of adapters, agarose gel size selection for fragments with average size about 186 bp, and PCR amplification to produce a DNA library of adapter-modified fragments. DNA sequencing was carried out using the Illumina/Solexa Genome Analyzer sequencing system at a concentration of 2 to 4 pM. Cluster amplification, linearization, blocking and sequencing primer reagents were provided in the Solexa Cluster Amplification kits and were used according to the manufacturer's specifications.

### Mapping the short reads to the genome and identifying peaks

Mapping the short reads is the same as reported Malone et al. (2011) [73]. The generated short reads were mapped onto the genome using SeqMap [89] allowing up to two mismatches between the short read and genome. The Illumina reads were aligned to TIGR version 6 of the rice genome [90]. The alignments were output in ELAND format. Only reads which mapped uniquely to the genome were retained. FindPeaks [91] was used to identify peaks with the mapped reads and the parameters used were the same as reported [73]. All the ChIP-Seq data were deposit to NCBI's Gene Expression Omnibus (http://www.ncbi.nlm.nih.gov/geo/) with the deposition number GSE27048 for genome-wide maps of chromatin state in rice endosperm.

## Supporting Information

Figure S1 H3 methylation status in *OsFIE2*-RNAi line NO9, *OsFIE2*-overexpression line 03A and wild type plants. Equal amounts of protein samples were loaded for Western blot analyses. The specific antibodies used are indicated on the right and the protein sample sources are shown on the top. When quantify the band intensity, the signals were normalized with the unmodified H3 band as control using the PDQUEST software.
(PDF)

Figure S2 Screenshot images of DNA enrichment profile at representative H3K27me3 binding sites. The chromatin used for the ChIP experiment was isolated from young endosperm before starch and storage protein synthesis. Gene's position in the chromatin is indicated by the scale line on the top of each panel. ChIP: DNA sample with immunoprecipitation for enrichment of the chromatin fragments associated with H3K27me3; Input: DNA sample without immunoprecipitation treatment. The sequence read members were normalized to ensure that the ChIP and the Input had identical read numbers over the entire genome. Therefore, the height of the graph in this figure directly correlates with the read number in the region to visually display the DNA enrichment. (A) Screenshot image of gene: LOC_Os04g35010. (B) Screenshot image of gene: LOC_Os05g28320. (C) Screenshot image of gene: LOC_Os07g11920. (D) Screenshot image of gene: LOC_Os07g11380. (E) Screenshot image of gene: LOC_Os07g11510. (F) Screenshot image of gene: LOC_Os07g11630. (G) Screenshot image of gene: LOC_Os07g10580. (H) Screenshot image of gene: LOC_Os10g30156.
(PDF)

Figure S3 Reverse-Transcription-PCR analysis of the expression of *OsFIE1* gene in *OsFIE2* RNAi lines. GAPDH was used as internal control. Same amount of cDNA template was used in each sample. Primers are listed in Table S3.
(PDF)

Table S1 Endosperm Specific and Nutrient Metabolic Genes Identified by H3K27me3 ChIP Enrichment.
(PDF)

Table S2 The list of H3K27me3 enriched peaks in rice endosperm.
(XLS)

Table S3 List of primers used in the study.
(PDF)

Table S4 List of antibodies used in the study.
(PDF)
