# Supplementary material for: Polycomb Group Gene OsFIE2 Regulates Rice (Oryza sativa) Seed Development and Grain Filling via a Mechanism Distinct from Arabidopsis
Source: PLoS Genet. 2013 Mar 7;9(3):e1003322. doi: 10.1371/journal.pgen.1003322 (PMC3591265; doi:10.1371/journal.pgen.1003322)
Supplement: Figure S1 — H3 methylation status in OsFIE2-RNAi line NO9, OsFIE2-overexpression line 03A and wild type plants. Equal amounts of protein samples were loaded for Western blot analyses. The specific antibodies used are indicated on the right and the protein sample sources are shown on the top. When quantify the band intensity, the signals were normalized with the unmodified H3 band as control using the PDQUEST software. (PDF) [file pgen.1003322.s001.pdf]

Figure S1

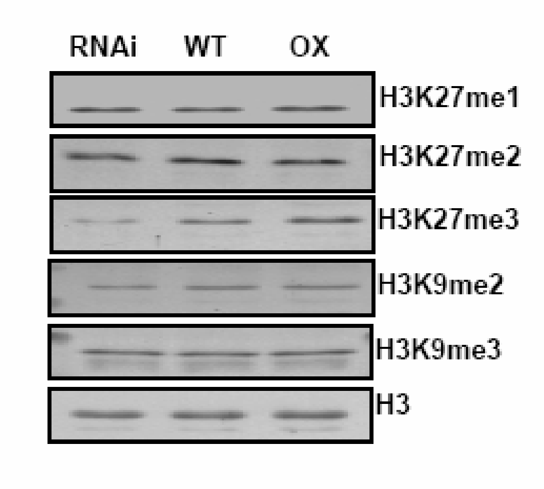

Figure S1. H3 methylation status in *OsFIE2*-RNAi line NO9, *OsFIE2*-overexpression line 03A and wild type plants. Equal amounts of protein samples were loaded for Western blot analyses. The specific antibodies used are indicated on the right and the protein sample sources are shown on the top. When quantify the band intensity, the signals were normalized with the unmodified H3 band as control using the PDQUEST software.
