# Supplementary material for: Polycomb Group Gene OsFIE2 Regulates Rice (Oryza sativa) Seed Development and Grain Filling via a Mechanism Distinct from Arabidopsis
Source: PLoS Genet. 2013 Mar 7;9(3):e1003322. doi: 10.1371/journal.pgen.1003322 (PMC3591265; doi:10.1371/journal.pgen.1003322)
Supplement: Figure S2 — Screenshot images of DNA enrichment profile at representative H3K27me3 binding sites. The chromatin used for the ChIP experiment was isolated from young endosperm before starch and storage protein synthesis. Gene's position in the chromatin is indicated by the scale line on the top of each panel. ChIP: DNA sample with immunoprecipitation for enrichment of the chromatin fragments associated with H3K27me3; Input: DNA sample without immunoprecipitation treatment. The sequence read members were normalized to ensure that the ChIP and the Input had identical read numbers over the entire genome. Therefore, the height of the graph in this figure directly correlates with the read number in the region to visually display the DNA enrichment. (A) Screenshot image of gene: LOC_Os04g35010. (B) Screenshot image of gene: LOC_Os05g28320. (C) Screenshot image of gene: LOC_Os07g11920. (D) Screenshot image of gene: LOC_Os07g11380. (E) Screenshot image of gene: LOC_Os07g11510. (F) Screenshot image of gene: LOC_Os07g11630. (G) Screenshot image of gene: LOC_Os07g10580. (H) Screenshot image of gene: LOC_Os10g30156. (PDF) [file pgen.1003322.s002.pdf]

Figure S2

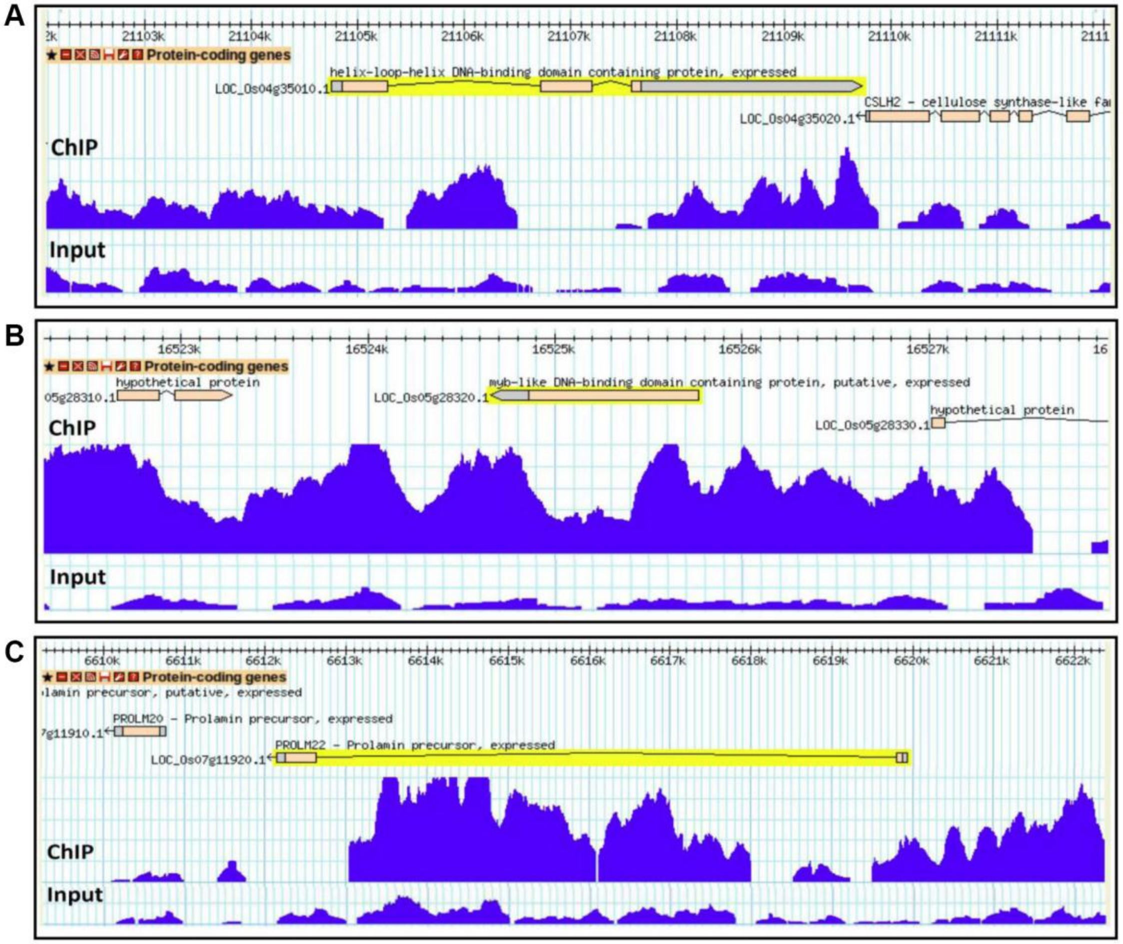
A
Protein-coding genes
helix-loop-helix DNA-binding domain containing protein, expressed
LOC_Os04g35010.1
CSLH2 - cellulose synthase-like fa
LOC_Os04g35020.1
ChIP
Input
B
Protein-coding genes
hypothetical protein
myb-like DNA-binding domain containing protein, putative, expressed
LOC_Os05g28320.1
hypothetical protein
LOC_Os05g28330.1
ChIP
Input
C
Protein-coding genes
PROLM20 - Prolamin precursor, expressed
PROLM22 - Prolamin precursor, expressed
LOC_Os07g11920.1
ChIP
Input

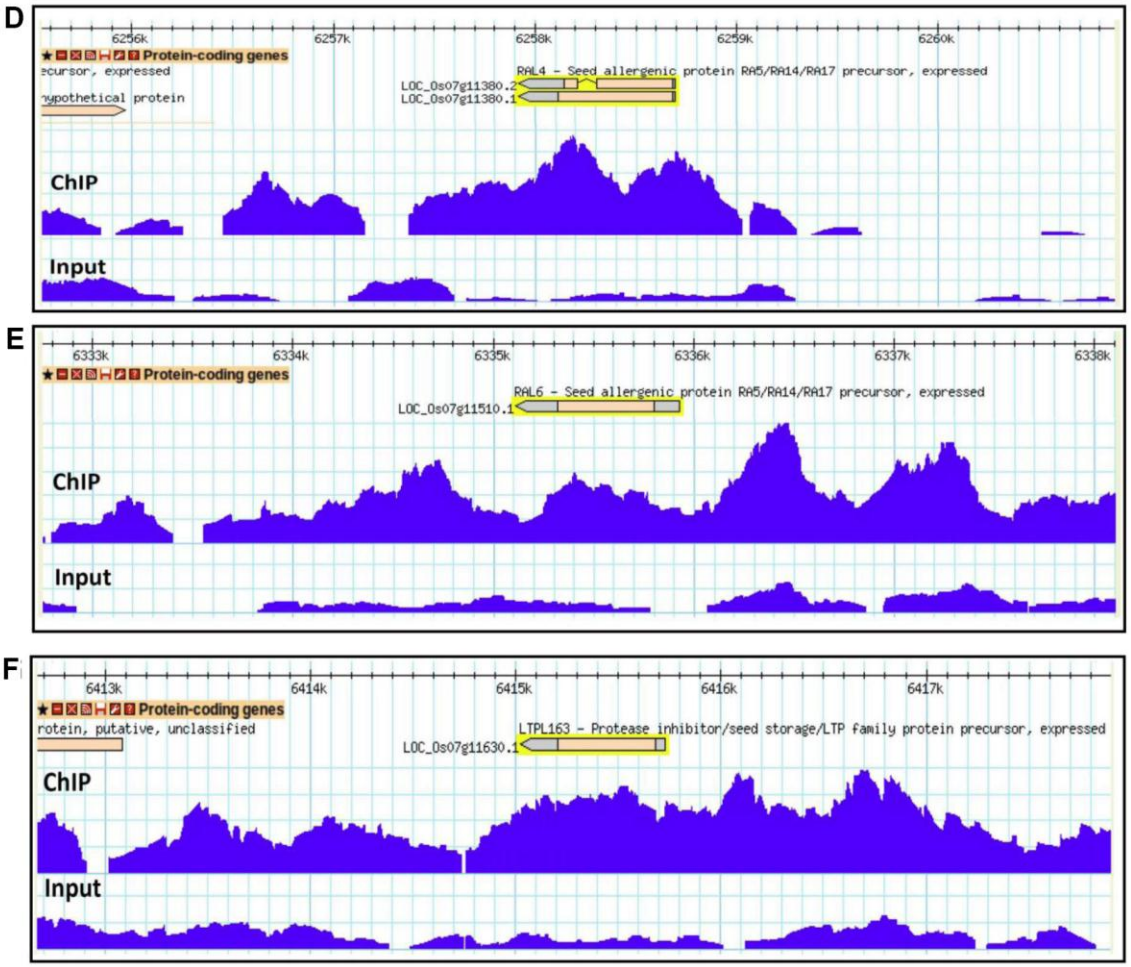
D
6256k
6257k
6258k
6259k
6260k
Protein-coding genes
RAL4 - Seed allergenic protein RA5/RA14/RA17 precursor, expressed
LOC_Os07g11380.2
LOC_Os07g11380.1
ChIP
Input
E
6333k
6334k
6335k
6336k
6337k
6338k
Protein-coding genes
RAL6 - Seed allergenic protein RA5/RA14/RA17 precursor, expressed
LOC_Os07g11510.1
ChIP
Input
F
6413k
6414k
6415k
6416k
6417k
Protein-coding genes
LTPL163 - Protease inhibitor/seed storage/LTP family protein precursor, expressed
LOC_Os07g11630.1
ChIP
Input

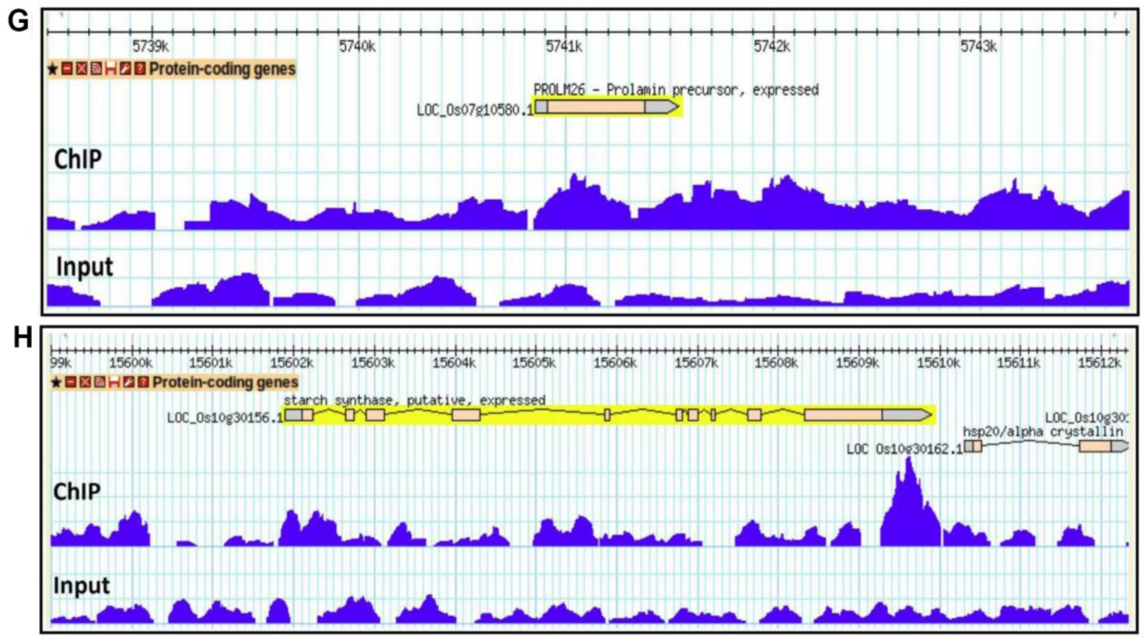

Figure S2. Screenshot images of DNA enrichment profile at representative H3K27me3 binding sites. The chromatin used for the ChIP experiment was isolated from young endosperm before starch and storage protein synthesis. Gene's position in the chromatin is indicated by the scale line on the top of each panel. ChIP: DNA sample with immunoprecipitation for enrichment of the chromatin fragments associated with H3K27me3; Input: DNA sample without immunoprecipitation treatment. The sequence read members were normalized to ensure that the ChIP and the Input had identical read numbers over the entire genome. Therefore, the height of the graph in this figure directly correlates with the read number in the region to visually display the DNA enrichment. **(A)** Screenshot image of gene: LOC_Os04g35010. **(B)** Screenshot image of gene: LOC_Os05g28320. **(C)** Screenshot image of gene: LOC_Os07g11920. **(D)** Screenshot image of gene: LOC_Os07g11380. **(E)** Screenshot image of gene: LOC_Os07g11510. **(F)** Screenshot image of gene: LOC_Os07g11630. **(G)** Screenshot image of gene: LOC_Os07g10580. **(H)** Screenshot image of gene: LOC_Os10g30156.
