# Supplementary material for: Polycomb Group Gene OsFIE2 Regulates Rice (Oryza sativa) Seed Development and Grain Filling via a Mechanism Distinct from Arabidopsis
Source: PLoS Genet. 2013 Mar 7;9(3):e1003322. doi: 10.1371/journal.pgen.1003322 (PMC3591265; doi:10.1371/journal.pgen.1003322)
Supplement: Figure S3 — Reverse-Transcription-PCR analysis of the expression of OsFIE1 gene in OsFIE2 RNAi lines. GAPDH was used as internal control. Same amount of cDNA template was used in each sample. Primers are listed in Table S3. (PDF) [file pgen.1003322.s003.pdf]

Figure S3

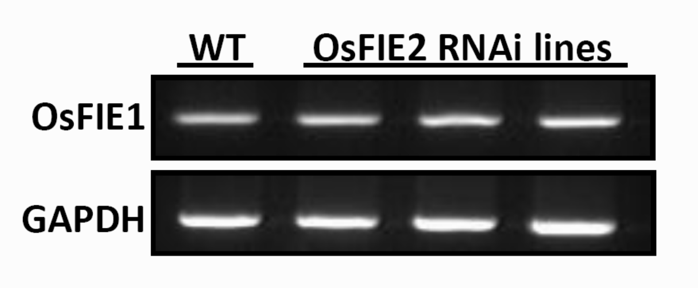

Figure S3. Reverse-Transcription-PCR analysis of the expression of *OsFIE1* gene in *OsFIE2* RNAi lines. GAPDH was used as internal control. Same amount of cDNA template was used in each sample. Primers are listed in Table S3.
