# Supplementary material for: Polycomb Group Gene OsFIE2 Regulates Rice (Oryza sativa) Seed Development and Grain Filling via a Mechanism Distinct from Arabidopsis
Source: PLoS Genet. 2013 Mar 7;9(3):e1003322. doi: 10.1371/journal.pgen.1003322 (PMC3591265; doi:10.1371/journal.pgen.1003322)
Supplement: Table S1 — Endosperm Specific and Nutrient Metabolic Genes Identified by H3K27me3 ChIP Enrichment. (PDF) [file pgen.1003322.s004.pdf]

Table S1. Endosperm Specific and Nutrient Metabolic Genes Identified by H3K27me3 ChIP Enrichment.

**Locus Number**	**Tissue Specificity**	**Gene Name**	**Enriched Region**	**Analysis of Algorithm**	**Start position**	**End position**	**False Discovery Rate**
LOC_Os01g11820	endosperm	expressed protein	Upstream	FindPeaks	6392689	6393189	FDR: 2.6848913E-5
LOC_Os02g09310	endosperm	cytochrome P450, putative	Upstream	FindPeaks	4789686	4790526	FDR: 0.0011761788
LOC_Os02g09670	endosperm	MYB protein, putative, expressed	Upstream	FindPeaks	4965306	4965733	FDR: 0.043232728
LOC_Os02g34370	endosperm	expressed protein	Upstream	FindPeaks	20553531	20555681	FDR: 0.0
LOC_Os02g36670	endosperm	hypothetical protein	Upstream	FindPeaks	22131376	22132401	FDR: 0.0032262774
LOC_Os02g46290	endosperm	invertase/pectin methylesterase inhibitor family protein, putative	Upstream	FindPeaks	28216717	28217191	FDR: 6.55308E-5
LOC_Os04g49720	endosperm	pectinesterase inhibitor domain containing protein, putative	Upstream	FindPeaks	29467221	29468351	FDR: 0.0
LOC_Os05g16160	endosperm	malonyl-CoA isoflavone 7-O-glucoside-6-O-malonyltransferase,	Upstream	FindPeaks	9135598	9136333	FDR: 3.9670253E-4
LOC_Os05g28830	endosperm	PMR5, putative, expressed	Upstream	FindPeaks	16839343	16839905	FDR: 6.55308E-5
LOC_Os05g42120	endosperm	heat shock protein, putative, expressed	Upstream	FindPeaks	24561970	24562865	FDR: 0.0
LOC_Os05g46150	endosperm	OsFBDUF28 - F-box and DUF domain containing protein	Upstream	FindPeaks	26703362	26703900	FDR: 0.0032262774
LOC_Os06g42910	endosperm	expressed protein	Upstream	FindPeaks	25782276	25783038	FDR: 0.0
LOC_Os07g17010	endosperm	chalcone synthase, putative	Upstream	FindPeaks	10015596	10015918	FDR: 0.0
LOC_Os08g03470	endosperm	MBTB15 - Bric-a-Brac, Tramtrack, Broad Complex BTB domain	Upstream	FindPeaks	1625149	1626073	FDR: 0.0032262774
LOC_Os08g04740	endosperm	expressed protein	Upstream	FindPeaks	2366880	2368036	FDR: 1.2576507E-4
LOC_Os09g14550	endosperm	RNA recognition motif containing protein, putative, expressed	Upstream	FindPeaks	8610386	8611307	FDR: 0.0
LOC_Os09g25890	endosperm	trehalose-6-phosphate synthase	Upstream	FindPeaks	15529204	15530268	FDR: 3.9670253E-4
LOC_Os10g04890	endosperm	expressed protein	Upstream	FindPeaks	2370834	2372200	FDR: 0.0
LOC_Os11g08220	endosperm	DEFL28 - Defensin and Defensin-like DEFL family	Upstream	FindPeaks	4310010	4310382	FDR: 0.043232728
LOC_Os11g11430	endosperm	OsIAA29 - Auxin-responsive Aux/IAA gene family	Upstream	FindPeaks	6351571	6351984	FDR: 3.9670253E-4
LOC_Os02g28580	endospermTF	expressed protein	Upstream	FindPeaks	16908139	16909135	FDR: 0.01937338
LOC_Os04g35010	endosperm TF	helix-loop-helix DNA-binding domain containing protein	Upstream	FindPeaks	21101993	21102904	FDR: 0.043232728
LOC_Os09g34880	endosperm TF	basic region leucine zipper domain containing protein	Upstream	FindPeaks	20324277	20324812	FDR: 0.043232728
LOC_Os09g38010	endosperm TF	no apical meristem protein, putative,	Upstream	FindPeaks	21904729	21906062	FDR: 0.0
LOC_Os01g03890	Nutrition	DUF260 domain containing protein, putative, expressed	Upstream	FindPeaks	1653889	1655768	FDR: 0.0
LOC_Os01g07850	Nutrition	glyoxalase family protein, putative, expressed	Upstream	FindPeaks	3775760	3777027	FDR: 2.6308867E-6
LOC_Os01g49660	Nutrition	reticulon domain containing protein, putative	Upstream	FindPeaks	28537133	28537781	FDR: 0.0032262774
LOC_Os01g68580	Nutrition	LTPL38 - Protease inhibitor/seed storage/LTP family protein	Upstream	FindPeaks	39833149	39835356	FDR: 0.0
LOC_Os01g68589	Nutrition	LTPL39 - Protease inhibitor/seed storage/LTP family protein	Upstream	FindPeaks	39835377	39836386	FDR: 0.0
LOC_Os02g14600	Nutrition	glutelin, putative, expressed	Upstream	FindPeaks	8055085	8055792	FDR: 0.01937338
LOC_Os02g56320	Nutrition	glycogen synthase 1, putative, expressed	Upstream	FindPeaks	34467168	34468703	FDR: 0.0
LOC_Os03g18130	Nutrition	asparagine synthetase, putative, expressed	Upstream	FindPeaks	10124005	10124519	FDR: 0.043232728
LOC_Os03g21080	Nutrition	guanine nucleotide exchange factor, putative, expressed	Upstream	FindPeaks	11998896	11999238	FDR: 0.043232728
LOC_Os03g24460	Nutrition	aminotransferase domain containing protein, putative,	Upstream	FindPeaks	13948766	13950830	FDR: 0.0032262774
LOC_Os03g63330	Nutrition	aspartokinase, chloroplast precursor, putative, expressed	Upstream	FindPeaks	35781791	35782147	FDR: 0.0
LOC_Os04g49980	Nutrition	late embryogenesis abundant group 1	Upstream	FindPeaks	29624425	29626020	FDR: 0.0

LOC_Os04g50970	Nutrition	seed specific protein Bn15D1B, putative, expressed	Upstream	FindPeaks	29989667	29990073	FDR: 0.043232728
LOC_Os05g39320	Nutrition	Thiamine pyrophosphate enzyme, C-terminal TPP binding	Upstream	FindPeaks	23001511	23002166	FDR: 6.55308E-5
LOC_Os05g47640	Nutrition	threonine synthase, chloroplast precursor, putative	Upstream	FindPeaks	27226721	27227326	FDR: 3.9670253E-4
LOC_Os06g01580	Nutrition	LTPL127 - Protease inhibitor/seed storage/LTP family protein	Upstream	FindPeaks	341345	341719	FDR: 3.9670253E-4
LOC_Os06g04200	Nutrition	starch synthase, putative, expressed	Upstream	FindPeaks	1763313	1764260	FDR: 0.0
LOC_Os06g13450	Nutrition	cystathionine beta-lyase, putative	Upstream	FindPeaks	7397462	7399808	FDR: 0.0
LOC_Os07g04910	Nutrition	reticulon domain containing protein, putative, expressed	Upstream	FindPeaks	2167465	2168475	FDR: 0.0
LOC_Os07g06660	Nutrition	glyoxalase family protein, putative, expressed	Upstream	FindPeaks	3248302	3249133	FDR: 0.0032262774
LOC_Os08g01760	Nutrition	dehydrogenase, putative, expressed	Upstream	FindPeaks	456023	456644	FDR: 0.043232728
LOC_Os08g36900	Nutrition	alpha-amylase precursor, putative, expressed	Upstream	FindPeaks	23331075	23331650	FDR: 3.9670253E-4
LOC_Os09g28400	Nutrition	alpha-amylase precursor, putative, expressed	Upstream	FindPeaks	17285839	17286349	FDR: 0.0032262774
LOC_Os10g26010	Nutrition	cystathionine gamma-synthase, putative, expressed	Upstream	FindPeaks	13392815	13394750	FDR: 0.0
LOC_Os10g33800	Nutrition	lactate/malate dehydrogenase, putative, expressed	Upstream	FindPeaks	17839380	17840744	FDR: 0.0
LOC_Os11g34780	Nutrition	globulin 2, putative	Upstream	FindPeaks	19908931	19910163	FDR: 1.2576507E-4
LOC_Os11g37280	Nutrition	LTPL68 - Protease inhibitor/seed storage/LTP family protein	Upstream	FindPeaks	21549315	21549785	FDR: 2.6848913E-5
LOC_Os01g06240	endosperm	protein kinase, putative, expressed	Within	FindPeaks	2961702	2962350	FDR: 0.008066991
LOC_Os01g58290	endosperm	OsSub9 - Putative Subtilisin homologue, expressed	Within	FindPeaks	33691445	33692229	FDR: 0.0032262774
LOC_Os01g58660	endosperm	LTPL29 - Protease inhibitor/seed storage/LTP family protein	Within	FindPeaks	33909249	33911042	FDR: 0.0
LOC_Os02g09190	endosperm	cytochrome P450, putative, expressed	Within	FindPeaks	4721375	4722351	FDR: 0.0032262774
LOC_Os02g09270	endosperm	cytochrome P450 71A1, putative	Within	FindPeaks	4776209	4776736	FDR: 6.55308E-5
LOC_Os02g09290	endosperm	cytochrome P450 71D10, putative, expressed	Within	FindPeaks	4784927	4785601	FDR: 0.008066991
LOC_Os02g24010	endosperm	hypothetical protein	Within	FindPeaks	13909898	13910421	FDR: 2.6E-5
LOC_Os02g29230	endosperm	conserved hypothetical protein	Within	FindPeaks	17352952	17353765	FDR: 0.01937338
LOC_Os02g34360	endosperm	conserved hypothetical protein	Within	FindPeaks	20549294	20551213	FDR: 0.0
LOC_Os02g48570	endosperm	peptide transporter PTR2, putative, expressed	Within	FindPeaks	29732804	29733551	FDR: 0.0011761788
LOC_Os03g17130	endosperm	bHelix-loop-helix transcription factor, putative, expressed	Within	FindPeaks	9512956	9513469	FDR: 0.01937338
LOC_Os03g29614	endosperm	myb-like DNA-binding domain containing protein, putative	Within	FindPeaks	16878390	16879071	FDR: 0.008066991
LOC_Os04g12639	endosperm	carboxyl-terminal proteinase, putative, expressed	Within	FindPeaks	6993795	6994670	FDR: 0.0
LOC_Os04g35000	endosperm	expressed protein	Within	FindPeaks	21100516	21101266	FDR: 6.55308E-5
LOC_Os05g07850	endosperm	receptor-like kinase, putative	Within	FindPeaks	4234341	4235113	FDR: 0.0
LOC_Os05g47820	endosperm	expressed protein	Within	FindPeaks	27349726	27350398	FDR: 0.01937338
LOC_Os05g50920	endosperm	transmembrane amino acid transporter protein, putative	Within	FindPeaks	29160936	29161515	FDR: 0.008066991
LOC_Os07g01700	endosperm	DEFL2 - Defensin and Defensin-like DEFL family, expressed	Within	FindPeaks	417112	418984	FDR: 0.0
LOC_Os07g17520	endosperm	conserved hypothetical protein	Within	FindPeaks	10350860	10352880	FDR: 0.0
LOC_Os08g16650	endosperm	hypothetical protein	Within	FindPeaks	10186609	10187483	FDR: 6.55308E-5
LOC_Os10g04900	endosperm	OsFBX364 - F-box domain containing protein, expressed	Within	FindPeaks	2384122	2386421	FDR: 0.0
LOC_Os10g07998	endosperm	nodulin, putative, expressed	Within	FindPeaks	4310312	4311338	FDR: 0.0
LOC_Os11g03270	endosperm	nucleoside-triphosphatase, putative	Within	FindPeaks	1200811	1202107	FDR: 1.2576507E-4
LOC_Os11g14880	endosperm	LTPL32 - Protease inhibitor/seed storage/LTP family protein	Within	FindPeaks	8367827	8368531	FDR: 0.0
LOC_Os12g08780	endosperm	flavin monooxygenase, putative, expressed	Within	FindPeaks	4514118	4514738	FDR: 0.0
LOC_Os12g28090	endosperm	expressed protein	Within	FindPeaks	16580214	16580755	FDR: 0.01937338

LOC_Os06g40330	endosperm TF	MYB family transcription factor	Within	FindPeaks	24010229	24011436	FDR: 0.0
LOC_Os10g25850	endosperm TF	nuclear transcription factor Y subunit, putative, expressed	Within	FindPeaks	13322384	13322963	FDR: 0.043232728
LOC_Os01g18860	Nutrition	S-adenosylmethionine synthetase, putative, expressed	Within	FindPeaks	10658710	10659768	FDR: 0.043232728
LOC_Os01g52250	Nutrition	starch synthase, putative, expressed	Within	FindPeaks	30029658	30030075	FDR: 3.9E-4
LOC_Os01g55630	Nutrition	glutelin, putative	Within	FindPeaks	32051748	32052917	FDR: 0.043232728
LOC_Os01g63810	Nutrition	starch binding domain containing protein, putative	Within	FindPeaks	37020968	37021639	FDR: 0.01937338
LOC_Os01g70700	Nutrition	conserved hypothetical protein	Within	FindPeaks	40927684	40928231	FDR: 1.2576507E-4
LOC_Os02g15090	Nutrition	glutelin, putative, expressed	Within	FindPeaks	8408517	8409569	FDR: 0.0
LOC_Os02g52700	Nutrition	alpha-amylase precursor, putative, expressed	Within	FindPeaks	32237372	32238065	FDR: 0.043232728
LOC_Os03g11660	Nutrition	cysteine synthase, putative, expressed	Within	FindPeaks	6084334	6085408	FDR: 0.008066991
LOC_Os03g41080	Nutrition	seed maturation protein PM23, putative, expressed	Within	FindPeaks	22828860	22829549	FDR: 0.043232728
LOC_Os03g42110	Nutrition	semialdehyde dehydrogenase, NAD binding domain containing	Within	FindPeaks	23436276	23436759	FDR: 0.008066991
LOC_Os03g46100	Nutrition	cupin domain containing protein, expressed	Within	FindPeaks	26068251	26069753	FDR: 0.0
LOC_Os04g44470	Nutrition	KUN1 - Kunitz-type trypsin inhibitor precursor, expressed	Within	FindPeaks	26138496	26138842	FDR: 0.008066991
LOC_Os04g46560	Nutrition	lactate/malate dehydrogenase, putative, expressed	Within	FindPeaks	27423157	27423547	FDR: 0.043232728
LOC_Os04g58720	Nutrition	anthranilate phosphoribosyltransferase, putative, expressed	Within	FindPeaks	34734430	34736426	FDR: 0.0011761788
LOC_Os05g04510	Nutrition	S-adenosylmethionine synthetase, putative, expressed	Within	FindPeaks	2090452	2090870	FDR: 0.01937338
LOC_Os05g29880	Nutrition	3-hydroxyacyl-CoA dehydrogenase, putative, expressed	Within	FindPeaks	17234000	17234430	FDR: 0.01937338
LOC_Os05g37450	Nutrition	starch binding domain containing protein, putative	Within	FindPeaks	21852286	21852920	FDR: 1.2576507E-4
LOC_Os05g39310	Nutrition	thiamine pyrophosphate enzyme, C-terminal TPP binding domain	Within	FindPeaks	22997130	22997881	FDR: 6.55308E-5
LOC_Os05g45720	Nutrition	starch synthase, putative, expressed	Within	FindPeaks	26431152	26431496	FDR: 0.0032262774
LOC_Os06g02040	Nutrition	late embryogenesis abundant group 1, putative, expressed	Within	FindPeaks	591652	593462	FDR: 0.0
LOC_Os06g12450	Nutrition	soluble starch synthase 2-3, chloroplast precursor, putative,	Within	FindPeaks	6750904	6751485	FDR: 0.01937338
LOC_Os06g35540	Nutrition	aminotransferase, classes I and II, domain containing protein	Within	FindPeaks	20725540	20726302	FDR: 2.6848913E-5
LOC_Os06g49970	Nutrition	alpha-amylase precursor, putative, expressed	Within	FindPeaks	30264503	30265965	FDR: 0.008066991
LOC_Os07g11310	Nutrition	LTPL166 - Protease inhibitor/seed storage/LTP family protein	Within	FindPeaks	6236698	6238140	FDR: 0.0
LOC_Os07g11410	Nutrition	RAL5 - Seed allergenic protein RA5/RA14/RA17 precursor	Within	FindPeaks	6274868	6276023	FDR: 2.6308867E-6
LOC_Os07g11510	Nutrition	RAL6 - Seed allergenic protein RA5/RA14/RA17 precursor	Within	FindPeaks	6333557	6335139	FDR: 0.0
LOC_Os07g11920	Nutrition	PROLM22 - Prolamin precursor, expressed	Within	FindPeaks	6613059	6616110	FDR: 0.0
LOC_Os07g49250	Nutrition	thiamine pyrophosphate enzyme, C-terminal TPP binding domain	Within	FindPeaks	29490574	29491339	FDR: 0.01937338
LOC_Os08g09200	Nutrition	aconitate hydratase protein, putative, expressed	Within	FindPeaks	5331601	5332298	FDR: 2.6308867E-6
LOC_Os08g29170	Nutrition	dehydrogenase, putative, expressed	Within	FindPeaks	17853210	17853727	FDR: 0.043232728
LOC_Os08g44530	Nutrition	dihydroxy-acid dehydratase, putative, expressed	Within	FindPeaks	28001765	28002545	FDR: 0.043232728
LOC_Os09g16650	Nutrition	legume lectins beta domain containing protein, putative	Within	FindPeaks	10203145	10203938	FDR: 0.008066991
LOC_Os09g28420	Nutrition	alpha-amylase precursor, putative, expressed	Within	FindPeaks	17302038	17302789	FDR: 0.043232728
LOC_Os09g28570	Nutrition	zinc-binding alcohol dehydrogenase domain-containing protein 2	Within	FindPeaks	17376381	17376616	FDR:
LOC_Os10g11140	Nutrition	phosphoglucomutase, putative, expressed	Within	FindPeaks	6188088	6188611	FDR: 0.0011761788
LOC_Os10g25930	Nutrition	cystathionine gamma-synthase, putative	Within	FindPeaks	13366508	13367009	FDR: 6.55308E-5
LOC_Os10g25950	Nutrition	cystathionine gamma-synthase, putative	Within	FindPeaks	13378393	13379211	FDR: 0.008066991
LOC_Os10g30156	Nutrition	starch synthase, putative, expressed	Within	FindPeaks	15609294	15610036	FDR: 0.0
LOC_Os10g30970	Nutrition	expressed protein	Within	FindPeaks	16095317	16096104	FDR: 1.2576507E-4

LOC_Os10g37340	Nutrition	cystathionine gamma-synthase, putative,	Within	FindPeaks	19930001	19930434	FDR: 0.0011761788
LOC_Os11g34660	Nutrition	LTPL98 - Protease inhibitor/seed storage/LTP family protein	Within	FindPeaks	19841421	19841700	FDR: 0.0011761788
LOC_Os01g28810	endosperm	expressed protein	Downstream	FindPeaks	16132384	16132970	FDR: 3.9670253E-4
LOC_Os01g48580	endosperm	ubiquitin-conjugating enzyme E2, putative	Downstream	FindPeaks	27861753	27862267	FDR: 0.0032262774
LOC_Os01g60050	endosperm	HEAT, putative	Downstream	FindPeaks	34728611	34729558	FDR: 0.01937338
LOC_Os02g25860	endosperm	glutelin, putative, expressed	Downstream	FindPeaks	15149387	15149707	FDR: 0.0032262774
LOC_Os02g43030	endosperm	integral membrane protein, putative, expressed	Downstream	FindPeaks	25902876	25903498	FDR: 0.043232728
LOC_Os02g46270	endosperm	hypothetical protein	Downstream	FindPeaks	28213190	28213832	FDR: 0.01937338
LOC_Os04g20774	endosperm	pumilio-family RNA binding repeat containing protein, expressed	Downstream	FindPeaks	11652005	11652948	FDR: 0.0
LOC_Os04g34510	endosperm	expressed protein	Downstream	FindPeaks	20721543	20723470	FDR: 3.9670253E-4
LOC_Os05g03870	endosperm	expressed protein	Downstream	FindPeaks	1722694	1723260	FDR: 0.01937338
LOC_Os06g04930	endosperm	expressed protein	Downstream	FindPeaks	2167125	2168790	FDR: 0.0
LOC_Os06g06260	endosperm	GDSL-like lipase/acylhydrolase, putative, expressed	Downstream	FindPeaks	2899904	2900360	FDR: 0.043232728
LOC_Os06g07700	endosperm	conserved hypothetical protein	Downstream	FindPeaks	3719372	3721122	FDR: 0.0
LOC_Os06g33490	endosperm	CAPIP1, putative	Downstream	FindPeaks	19505796	19506767	FDR: 1.2576507E-4
LOC_Os06g51084	endosperm	1,4-alpha-glucan-branching enzyme, chloroplast precursor, putative	Downstream	FindPeaks	30894028	30895075	FDR: 0.0032262774
LOC_Os06g51140	endosperm	ZOS6-11 - C2H2 zinc finger protein, expressed	Downstream	FindPeaks	30931468	30933501	FDR: 0.0
LOC_Os07g17460	endosperm	OsFBL36 - F-box domain and LRR containing protein	Downstream	FindPeaks	10301577	10303273	FDR: 0.0
LOC_Os07g31470	endosperm	MYB family transcription factor,	Downstream	FindPeaks	18655567	18656659	FDR: 0.0
LOC_Os09g16260	endosperm	expressed protein	Downstream	FindPeaks	9917993	9918828	FDR: 0.0
LOC_Os10g04980	endosperm	OsFBX365 - F-box domain containing protein, expressed	Downstream	FindPeaks	2417211	2419154	FDR: 0.0
LOC_Os11g45360	endosperm	DEFL15 - Defensin and Defensin-like DEFL family	Downstream	FindPeaks	26964268	26964719	FDR: 0.01937338
LOC_Os12g39910	endosperm	hypothetical protein	Downstream	FindPeaks	24625269	24627050	FDR: 0.0
LOC_Os05g28320	endosperm TF	myb-like DNA-binding domain containing protein, putative	Downstream	FindPeaks	16521019	16523185	FDR: 0.0
LOC_Os09g31390	endosperm TF	transcription factor, putative, expressed	Downstream	FindPeaks	18866552	18867187	FDR: 0.043232728
LOC_Os12g39400	endosperm TF	ZOS12-09 - C2H2 zinc finger protein, expressed	Downstream	FindPeaks	24201010	24201961	FDR: 6.55308E-5
LOC_Os01g46600	Nutrition	seed maturation protein PM41, putative, expressed	Downstream	FindPeaks	26530041	26530703	FDR: 0.043232728
LOC_Os01g51210	Nutrition	uncharacterized protein PA4923, putative, expressed	Downstream	FindPeaks	29435593	29435976	FDR: 0.008066991
LOC_Os01g55540	Nutrition	aminotransferase, classes I and II, domain containing protein	Downstream	FindPeaks	31995355	31996938	FDR: 0.0
LOC_Os02g03690	Nutrition	beta-amylase, putative, expressed	Downstream	FindPeaks	1549495	1549864	FDR: 0.008066991
LOC_Os02g06720	Nutrition	WD domain containing protein, putative, expressed	Downstream	FindPeaks	3360761	3361599	FDR: 0.01937338
LOC_Os02g24020	Nutrition	dihydrodipicolinate reductase, putative, expressed	Downstream	FindPeaks	13909898	13910421	FDR: 2.6848913E-5
LOC_Os02g32660	Nutrition	1,4-alpha-glucan-branching enzyme, chloroplast precursor, putative	Downstream	FindPeaks	19348075	19348812	FDR: 0.043232728
LOC_Os03g14630	Nutrition	LTPL106 - Protease inhibitor/seed storage/LTP family protein	Downstream	FindPeaks	7941908	7943581	FDR: 0.0
LOC_Os03g19390	Nutrition	TENA/THI-4 family protein, putative, expressed	Downstream	FindPeaks	10907870	10908740	FDR: 0.0032262774
LOC_Os03g31360	Nutrition	glutelin, putative, expressed	Downstream	FindPeaks	17865337	17866016	FDR: 0.0
LOC_Os03g50510	Nutrition	threonine dehydratase biosynthetic, chloroplast precursor, putative	Downstream	FindPeaks	28838192	28839680	FDR: 0.0032262774
LOC_Os03g56900	Nutrition	reticulon domain containing protein	Downstream	FindPeaks	32392898	32394125	FDR: 0.0432
LOC_Os03g58480	Nutrition	seed specific protein Bn15D14A, putative, expressed	Downstream	FindPeaks	33308724	33309340	FDR: 0.01937338
LOC_Os04g08270	Nutrition	pullulanase precursor, putative, expressed	Downstream	FindPeaks	4415018	4415579	FDR: 0.043232728
LOC_Os04g46370	Nutrition	L-asparaginase precursor protein, putative, expressed	Downstream	FindPeaks	27307809	27308368	FDR: 3.9670253E-4

LOC_Os05g06300	Nutrition	3-hydroxyacyl-CoA dehydrogenase, putative, expressed	Downstream	FindPeaks	3217579	3218122	FDR: 0.043232728
LOC_Os05g49800	Nutrition	ketol-acid reductoisomerase, chloroplast precursor, putative	Downstream	FindPeaks	28515099	28516096	FDR: 0.01937338
LOC_Os07g11320	Nutrition	RAL1 - Seed allergenic protein RA5/RA14/RA17 precursor	Downstream	FindPeaks	6238361	6238703	FDR: 3.9670253E-4
LOC_Os07g11330	Nutrition	RAL2 - Seed allergenic protein RA5/RA14/RA17 precursor	Downstream	FindPeaks	6240518	6241350	FDR: 0.0
LOC_Os07g11360	Nutrition	RAL3 - Seed allergenic protein RA5/RA14/RA17 precursor	Downstream	FindPeaks	6251780	6252459	FDR: 0.008066991
LOC_Os07g11380	Nutrition	RAL4 - Seed allergenic protein RA5/RA14/RA17	Downstream	FindPeaks	6256457	6257160	FDR: 0.0
LOC_Os07g11630	Nutrition	LTPL163 - Protease inhibitor/seed storage/LTP family protein	Downstream	FindPeaks	6413026	6413834	FDR: 1.2576507E-4
LOC_Os07g12090	Nutrition	LTPL165 - Protease inhibitor/seed storage/LTP family protein	Downstream	FindPeaks	6743386	6743918	FDR: 0.01937338
LOC_Os08g36910	Nutrition	alpha-amylase precursor, putative, expressed	Downstream	FindPeaks	23341766	23342310	FDR: 3.9670253E-4
LOC_Os08g40930	Nutrition	Alpha amylase, catalytic domain containing protein	Downstream	FindPeaks	25887090	25887788	FDR: 3.9670253E-4
LOC_Os09g10620	Nutrition	seed maturation protein LEA 4, putative	Downstream	FindPeaks	5789631	5789971	FDR: 0.01937338
LOC_Os09g37120	Nutrition	pyridoxal-dependent decarboxylase protein, putative, expressed	Downstream	FindPeaks	21406580	21407047	FDR: 0.01937338
LOC_Os10g40200	Nutrition	aminotransferase domain containing protein, putative	Downstream	FindPeaks	21454583	21455235	FDR: 0.0032262774
LOC_Os12g43630	Nutrition	lactate/malate dehydrogenase, putative, expressed	Downstream	FindPeaks	27058808	27060116	FDR: 0.0
LOC_Os02g45770	*This gene is not endosperm specific	OsMADS6- MADS box family protein	Upstream	FindPeaks	27870106	27871129	FDR: 0.0

Note: We used rice expression profile data in RICE GE data base to identify the endosperm specific genes by using the below mentioned criteria.

1. Endosperm has the highest expression among the 13 tissues we downloaded from riceGE (http://signal.salk.edu/cgi-bin/RiceGE) and the signal intensity is >500. This is to confirm genes are expressed in endosperm.
2. All the other tissues have intensity < 500. This is to confirm genes are not expressed in other tissues.
3. Endosperm signal intensity is 10 times higher than other tissues.
