# Supplementary material for: Polycomb Group Gene OsFIE2 Regulates Rice (Oryza sativa) Seed Development and Grain Filling via a Mechanism Distinct from Arabidopsis
Source: PLoS Genet. 2013 Mar 7;9(3):e1003322. doi: 10.1371/journal.pgen.1003322 (PMC3591265; doi:10.1371/journal.pgen.1003322)
Supplement: Table S3 — List of primers used in the study. (PDF) [file pgen.1003322.s006.pdf]

Table S3. List of primers used in the study

Primer Name	Sequence
61TAPF	CACCATGGCGAAGCTGGGGCC
61TAPR	TCTGGCATTGCCATGGTCTGTCT
61RNAiF	CACCGCCTTCGCTCATCATTTCTGC
61RNAiR	CTTTTCCTTCACGGTTGCCTATTG
OsFIE2-RTF	TTTTAACTGCTCGGCTGACT
OsFIE2-RTR	TCATGCTTTTGGATGGTCC
OsEMF2-RTF	AAAGGGTACTAGCGGATGGC
OsEMF2-RTR	GACTGTGGTTCCAGAGTTTGAC
OsEZ1-RTF	GCACGAGTATCTTGGCGAGTA
OsEZ1-RTR	TGATGACCTTGGCGTAGCA
OsEZ3-RTF	ATTACCGCTATGGACCTGACC
OsEZ3-RTR	TGGAGCTGCTGTTGGACTATC
OsFIE1-RTF	GCAAAGTTGGTGCCATTAGAGGG
OsFIE1-RTR	CAGTGGCACAGTTGATGACTCGAA
OsFIE2-pTRG- BamHI-F	CGGGATCCATGGCGAAGCTGGGGCCG
OsFIE2-pTRG- XhoI-R	CCGCTCGAGTCATGCTTTTGGATGGTC
OsEZ3-PBT- EcoRI-F	CGGAATTCCATGGCGTCGTCCTCGTCC
OsEZ3-PBT- BamHI-R	CGGGATCCCTATCTAGCAACTTTGTG
OsAGPS2bf	AACAATCGAAGCGCGAGAAA
OsAGPS2br	GCCTGTAGTTGGCACCCAGA
Glutelin A1 Os01g55690f	GTTTGGATGAGACCTTTTG
Glutelin A1 Os01g55690r	CTAAGAGGATTCTGCCGCA
23 Globulin Os05g41970f	ATGGCTAGCAAGGTCGTC
23 Globulin Os05g41970r	CACTGCTGGGGCTCAACC
29 Prolamine17 Os06g31070f	TGCCAGCAGCAGCCGTTT
29 Prolamine17 Os06g31070r	GGGCTCGCCAACAAAGCC
35 Alpha-amylase Os07g11650f	ATGGCATTGGCATCAGACA
35 Alpha-amylase Os07g11650r	ATGGGGCAGCCAAAGAA
36 Prolamin Os07g10570f	TTTGCTCTCCTTGCTATT
36 Prolamin Os07g10570r	GCACTATAGTAGTTAGGG
37 Prolamin14 Os05g26377f	TTTGCTCTCCTTGCTATT
37 Prolamin14 Os05g26377r	TTATATGGGCTAAGCACC
ChIP 04g35010 F	GTGTCTCCTCCTAGAACAATAAATC
ChIP 04g35010 R	CCCCTCCTATAATAACGAATCTG
04g35010 RT F	AGAGCGGAGGAAGAAGATGA
04g35010 RT R	AATGGCCTCCCCAACAAT
