# Supplementary material for: Polycomb Group Gene OsFIE2 Regulates Rice (Oryza sativa) Seed Development and Grain Filling via a Mechanism Distinct from Arabidopsis
Source: PLoS Genet. 2013 Mar 7;9(3):e1003322. doi: 10.1371/journal.pgen.1003322 (PMC3591265; doi:10.1371/journal.pgen.1003322)
Supplement: Table S4 — List of antibodies used in the study. (PDF) [file pgen.1003322.s007.pdf]

Table S4. List of antibodies used in the study

**Antibody**	**Company**	**Catalog number**
Peroxidase anti peroxidase (PAP)	Sigma	P1291
Biotinylated calmodulin (CAM)	Calbiochem	208697
Anti-H3 monomethyl-lysine 27 (H3K27me1)	Diagenode	pAb-045-050
Anti-H3 dimethyl-lysine 27 (H3K27me2)	Millipore	07-452
Anti-H3 trimethyl-lysine 27 (H3K27me3)	Millipore	07-449
Anti-H3 dimethyl-lysine 9 (H3K9me2)	Millipore	07-212
Anti-H3 trimethyl-lysine 9 (H3K9me3)	Millipore	07-523
Anti-histone H3 (H3)	Abcam	Ab1791
